# *Toxoplasma gondii* ROP18 inhibits human glioblastoma cell apoptosis through a mitochondrial pathway by targeting host cell P2X1

**DOI:** 10.1186/s13071-019-3529-1

**Published:** 2019-06-04

**Authors:** Li-Juan Zhou, Min Chen, Santhosh Puthiyakunnon, Cheng He, Jing Xia, Cynthia Y. He, Sheng-Qun Deng, Hong-Juan Peng

**Affiliations:** 1 Department of Pathogen Biology, Guangdong Provincial Key Laboratory of Tropical Disease Research, School of Public Health, Southern Medical University, Guangzhou, 510515 Guangdong People’s Republic of China; 2 Department of Biological Sciences, National University of Singapore, Singapore, Singapore

**Keywords:** *Toxoplasma gondii*, ROP18, Mitochondria, Apoptosis, P2X1, ATP

## Abstract

**Background:**

Apoptosis plays a critical role in the embryonic development, homeostasis of immune system and host defense against intracellular microbial pathogens. Infection by the obligate intracellular pathogen *Toxoplasma gondii* can both inhibit and induce host cell apoptosis; however, the parasitic factors involved remain unclear. The *T. gondii* virulence factor ROP18 (*Tg*ROP18) has been reported to regulate host cell apoptosis; nevertheless, results for this regulation have been rarely reported or have provided contradictory findings. Human purinergic receptor 1 (P2X1) is an ATP-gated ion channel that responds to ATP stimulation and functions in cell apoptosis mediation. The precise roles of *Tg*ROP18 in *T. gondii* pathogenesis, and the relationship between *Tg*ROP18 and host P2X1 in host cell apoptosis are yet to be revealed.

**Methods:**

Apoptosis rates were determined by flow cytometry (FCM) and TUNEL assay. The interaction between *Tg*ROP18 and the host P2X1 was measured by fluorescence resonance energy transfer (FRET) and co-immunoprecipitation (co-IP) assay. Calcium influx and mitochondrial membrane depolarization were determined by FCM after JC-1 staining. The translocation of cytochrome C (Cyt C), Bax and Bcl2 proteins, expression of the apoptotic proteins PARP and caspase activation were detected by western blotting.

**Results:**

The apoptosis rates of glial or immune cells (human SF268, mouse RAW264.7 and human THP-1 cells) infected by any *T. gondii* strain (RH-type I, ME49-type II and VEG-type III) were significantly inhibited compared with their uninfected controls. *Tg*ROP18 inhibited ATP-induced apoptosis of SF268 with P2X1 expression, but had no effect on RAW264.7 or THP-1 cells without detectable P2X1 expression. It was further identified that *Tg*ROP18 interacted with P2X1, and overexpression of ROP18 in COS7 cells significantly inhibited cell apoptosis mediated by P2X1. Moreover, *Tg*ROP18 also inhibited P2X1-mediated Ca^2+^ influx, translocation of cytochrome C from the mitochondria to the cytosol, and ATP-triggered caspase activation.

**Conclusions:**

*Toxoplasma gondii* infection inhibits ATP-induced host cell apoptosis, regardless of strain virulence and host cell lines. *Tg*ROP18 targets the purinergic receptor P2X1 of the SF268 human neural cells and inhibits ATP-induced apoptosis through the mitochondrial pathway, suggesting a sensor role for the host proapoptotic protein P2X1 in this process.

**Electronic supplementary material:**

The online version of this article (10.1186/s13071-019-3529-1) contains supplementary material, which is available to authorized users.

## Background

*Toxoplasma gondii*, an obligate intracellular protozoan, can infect humans and nearly all warm-blooded animals [1]. It is estimated that 30% of the world human population is infected with *T. gondii* [2]. *Toxoplasma gondii* infection shows no or mild symptoms in immune competent hosts; however, the symptoms may be severe in immunocompromised patients and after congenital infections [2]. Based on their acute virulence in the mouse model, *T. gondii* strains are categorized into the highly virulent type Ӏ (RH) strain with a lethal dose (LD) of one parasite, and non-virulent type II (ME49, PLK) and type III (CEP) strains with an LD50 of more than 1000 parasites [3, 4].

*Toxoplasma gondii* infection can both inhibit and induce host cell apoptosis. These opposing effects might involve complicated factors that modulate the finely balanced interaction between the parasite and the pro- and anti-apoptotic signals of the host, such as the host cell type, the virulence of *T. gondii* and others [5]. For example, tachyzoites of the RH strain promote apoptosis of mouse neural stem cells [6], while inhibiting apoptosis of human leukemic, THP-1 and Jurkat cells [7, 8]. Apoptosis of trophoblast cells can be induced by ME49 infection, whereas it is inhibited by RH infection [9]. *Toxoplasma gondii* ROP18 is a Ser/Thr kinase secreted by the rhoptries into the PV and host cytosol during invasion [10]. Among the laboratory strains that infect mice, type I strains avoid the accumulation of host immunity-related GTPases (IRGs) on the parasitophorous vacuole (PV) to protect the parasite from clearance, which is partly attributed to the expression of *Tg*ROP18 [11]. *Tg*ROP18 can degrade host cell proteins activating transcription factor 6 beta (ATF6β) or transcription factor P65 and help the parasite resist the host innate immune response [12, 13]. Moreover, ROP18 has also been reported to induce apoptosis of mouse N2a neural cells [14] and inhibit the apoptosis of the 293T human epithelial cells induced by actinomycin D (act D) [15]. However, the precise roles of *Tg*ROP18 and its significance to *T. gondii* pathogenesis are yet to be established.

Apoptosis can be triggered by either a physiologic or a pathologic stimulus, exhibiting cytoplasmic shrinkage, chromatin condensation, nuclear fragmentation, plasma membrane blebbing, and finally formation of apoptotic bodies which are efficiently absorbed by neighboring cells with phagocytic activity and degraded within lysosomes [16]. It plays a critical role not only in development and homeostasis but also in host defense against microbial infection [17, 18], particularly in clearance of intracellular microbial pathogens [19]. Therefore, for their survival, obligate intracellular pathogens like *T. gondii* have evolved many ways to interfere with the highly regulated host cell apoptotic pathways [20, 21].

Both immune response and apoptosis are triggered and regulated by signaling molecules. One of the signaling molecules is adenosine triphosphate (ATP). Inside a cell, ATP serves as a major energy source, whereas outside of the cell, ATP functions as a potent autocrine and paracrine signaling molecule for immune response [22–25]. For example, extracellular ATP can induce apoptosis in the human neuroblastoma cell line SH-SY5Y by decreasing expression of the anti-apoptotic protein Bcl2 and increasing expression of the proapoptotic protein Bax [26].

ATP signaling is mediated by cell-surface P2 purinergic receptors, which consist of two families of receptors, P2X and P2Y receptors [24, 25]. The former are cation channels gated by extracellular ATP, and the latter G-protein-coupled metabotropic receptors. P2X receptors are homotrimeric and heterotrimeric proteins assembled from seven types of subunits, P2X1–7 [27]. P2X1 is formed with a trimeric assembly of the subunits with two transmembrane regions, the intracellular amine and the carboxyl-terminal region, and a large extracellular ligand binding loop [28]. P2X1 has also been reported to be phosphorylated at the C-terminus, and a conformational change in P2X1 is required for coordination of ATP action [29]. Immunohistochemistry and functional studies have shown that P2X1 is expressed predominantly on the membrane of smooth muscle cells, blood cells and neurons [30–32]. P2X1 activation results in cell apoptosis characterized by Na^+^ and Ca^2+^ influx and K^+^ efflux, which leads to depolarization of the plasma membrane [32]. The cell apoptosis rate is increased in mouse thymocytes overexpressing P2X1, indicating that P2X1 is a proapoptotic protein [33]. Whether P2X1 is a target protein in the modulation of host cell apoptosis by *T. gondii* is not known.

In this study, we found that *T. gondii* infection inhibited the ATP-induced apoptosis of SF268, THP-1 and RAW264.7 cells, regardless of strain virulence and host cell lines tested. Furthermore, we showed that *Tg*ROP18 targeted the purinergic receptor P2X1 of the human neural cells and inhibited ATP-induced apoptosis through the mitochondrial pathway.

## Methods

### Reagents

The apoptosis inducer ATP was purchased from Sigma-Aldrich (St. Louis, USA), the P2X1 antagonist NF449 from Tocris Bioscience (Minneapolis, USA), Fluo-4 AM from Dojindo Laboratories (Mashiki-machi, Japan), cell mitochondria isolation kit from Beyotime Biotechnology (Beijing, China) and Lipofectamine® 3000 transfection kit from Thermo Fisher Scientific (Waltham, USA). An isothiocyanate (FITC)-Annexin V/propidium iodide (PI) apoptosis detection kit, TUNEL *in situ* cell death detection kit and JC-1 mitochondria membrane potential detection kit were purchased from KeyGen (Nanjing, China); protein A-agarose immunoprecipitation reagent and normal rabbit IgG from Santa Cruz Biotechnology (California, USA); HA-tag rabbit mAb, HA-tag mouse mAb, β-actin rabbit mAb, caspase-3 rabbit mAb, caspase-7 rabbit mAb, caspase-9 rabbit mAb and PARP rabbit mAb from Cell Signaling Technology (Boston, USA); Bax rabbit mAb, Bcl2 rabbit mAb, Cyt C rabbit mAb, P2X1 rabbit pAb and cytochrome C oxidase IV (COXIV) rabbit mAb from Abcam (Cambridge, UK); and DDDDK-Tag Mouse mAb from Abclonal (Boston, USA).

### Cell culture

The human monocyte/macrophage cell line THP-1 (ATCC, Virginia, USA) and human glioblastoma cell line SF268 (ATCC) were propagated in Roswell Park Memorial Institute 1640 medium (RPMI 1640; Thermo Fisher Scientific) supplemented with 10% (v/v) fetal bovine serum (FBS; Thermo Fisher Scientific, Massachusetts, USA), 100 U/ml penicillin and 100 µg/ml streptomycin (Thermo Fisher Scientific, Massachusetts, USA) at 37 °C and 5% CO_2_. The mouse monocyte/macrophage cell line RAW264.7 (ATCC), the *Cercopithecus aethiops* kidney fibroblast cell line COS7 (ATCC), and the human foreskin fibroblast cell line HFF (ATCC) were cultured in Dulbecco’s modified Eagle medium (DMEM; Thermo Fisher Scientific) supplemented with 10% (v/v) FBS, 100 U/ml penicillin and 100 µg/ml streptomycin at 37 °C and 5% CO_2_.

### *Toxoplasma gondii* culture, cell infection and induction of apoptosis

Type Ӏ strain RH, RH-Δ*rop18*, RH-ROP18-GFP-Flag, type II strain ME49 and type III strain VEG were cultured in HFFs in DMEM supplemented with 1% (v/v) FBS, 100 U/ml penicillin and 100 µg/ml streptomycin at 37 °C and 5% CO_2_. Three to five days later, when most of the cells were ready to be ruptured by the tachyzoites, the cells were scraped and harvested by passing through a syringe multiple time. The tachyzoites were then purified by passing them through a 3-µm filter (Whatman, Kent, UK), pelleting at 2500× *rpm* for 10 min and resuspending in DMEM. The tachyzoites were counted with a hemocytometer.

Before infection, THP-1 were cultured in the presence of 100 ng/ml phorbol 12-myristate 13-acetate (PMA; Abcam) for 24 h for differentiation into macrophages as described previously [34]. THP-1, RAW264.7 and SF268 cells were infected with RH, ME49 and VEG strains at a multiplicity of infection (MOI) of 3 and incubated for 2 or 22 h at 37 °C in 5% CO_2_. Before induction of apoptosis, cells were washed with phosphate buffered saline (PBS) to remove non-adherent parasites, and then the THP-1 cells, SF268 cells and RAW264.7 cells were all treated with 1 mg/ml ATP for 4 or 6 h, and the apoptotic level of the cells was analyzed by the AnnexinV-FITC/PI and TUNEL assay.

RH-wild type and RH-Δ*rop18* tachyzoites were separated from the host cells by centrifugation, then the SF268, THP-1 and RAW264.7 cells were infected with the RH-wild type and RH-*Δrop18* tachyzoites at MOI = 13. Cells were washed with PBS at 12 h post-infection and then treated with 1 mg/ml ATP for an additional 12 h to induce apoptosis. The apoptotic levels of the cells were analyzed by AnnexinV-FITC/PI assay.

### Flow cytometry detection of apoptosis with Annexin V-FITC/PI staining

The apoptotic levels of RAW264.7, THP-1 and SF268 cells were determined with annexin V- FITC/PI kit. Cells were briefly digested with 0.25% trypsin (Thermo Fisher Scientific) and washed twice with cold PBS. Then, they were resuspended in 500 μl of binding buffer, 5 μl of annexin V- FITC and 5 μl of PI and incubated at room temperature for 15 min in the dark. Annexin V-FITC binds to phosphatidylserine on the outer membrane of apoptotic cells, while PI enters and stains cells with compromised membrane integrity and then binds to and labels DNA. A flow cytometer (BD FACSCalibur; BD Biosciences, New Jersey, USA) was used for data collection, and FlowJo software for data analysis. The FSC-H/FSC-A dot plot was used to exclude clumped cells to select single cells. The cells in the annexin V-FITC^+^/PI^−^, annexinV-FITC^+^/PI^+^ and annexin V-FITC^-^/PI^+^ quadrants were counted as apoptotic cells.

### TUNEL assay

*In situ* labeling of fragmented DNA was performed using the terminal deoxynucleotidyl transferase dUTP nick end labeling (TUNEL) method following the manufacturer’s protocol. RAW264.7, THP-1 and SF268 cells were fixed with 4% formaldehyde and then permeabilized with 0.1% Triton-X-100 in 10 mM PBS. After washing 3 times with PBS, the cells were incubated in biotin-labeled dUTP solution for 60 min at 37 °C. After washing 3 times with PBS, the cells were incubated in streptavidin-fluorescein solution for 30 min at 37 °C. Then, the stained cells and total cells were calculated under a fluorescence microscope (Nikon, Tokyo, Japan), and the mean percentage of the stained cells among the total cells was calculated from 10 representative fields per coverslip. Finally, the mean percentage was calculated from the quadruplets in each treatment group. Four repetitive experiments were performed for statistical analysis.

### Fluorescence resonance energy transfer (FRET) experiment

COS7 cells were cultured in plates the day before transfection. pECFP-N1-ROP18 and pEYFP-C1-P2X1 were co-transfected into COS7 cells for the experimental group. pECFP-N1 and pEYFP-C1 were co-transfected into COS7 cells as a negative control and pEYFP-CFP was transfected into COS7 cells as a positive control [35]. Forty-eight hours post-transfection, the cells were fixed in 4% paraformaldehyde at 37 °C for 30 min and then washed twice with PBS. The FRET efficiency of the different transfection groups was measured under a confocal laser scanning microscope (FLUOVIEW FV1000; Olympus, Tokyo, Japan) [36]. Ten fields were evaluated, and four repetitive experiments performed independently for statistical analysis.

### Co-immunoprecipitation analysis

SF268 and COS7 cells were grown in T75 flasks to 100% confluence. SF268 cells were infected with or without the RH-ROP18-GFP-Flag strain for 36 h. COS7 cells were transfected with pcDNA3.1-P2X1-HA (or pcDNA3.1-P2X1Δ339-399-HA) individually or together with pcDNA3.1-ROP18-3×Flag for 48 h. The cells were washed 3 times with PBS and lysed using cell lysis buffer (Beyotime) with 1 mM phenylmethanesulfonyl fluoride (Dingguo Changsheng Biotechnology, Beijing China). Cell lysates were centrifuged at 14,000×*g* for 10 min at 4 °C. The supernatants were harvested and incubated with the primary antibody anti-HA antibody (3724S, CST), anti-FLAG antibody (F1804, Sigma Aldrich) or rabbit normal IgG for 2 h at 4 °C with gentle rotation. Then, protein A-agarose (Santa Cruz Biotechnology) was added to the mix and incubated overnight at 4 °C with gentle rotation. The beads were collected by centrifugation at 500×*g* for 5 min at 4 °C and then resuspended in SDS-PAGE sample buffer (TransGen, Beijing, China). The samples were boiled, loaded onto the gels for SDS-PAGE and then analyzed by Western blotting (WB) [37].

### Observation of the effect of *Tg*ROP18 overexpression on ATP-induced host cell apoptosis

COS7 cells were transfected with pcDNA3.1-ROP18-3×Flag individually or together with pcDNA3.1-P2X1-HA. At 48 h post-transfection, the COS7 cells were treated with 60 µg/ml ATP for 24 h to induce apoptosis. SF268 cells were treated with 4 µM NF449 (P2X1 receptor antagonist) for 2 h and then subjected to 1 mg/ml ATP treatment for 12 h. The normal control cells were treated with neither NF449 nor ATP. The apoptosis rate of the cells was analyzed by FCM after annexin V-FITC/PI staining.

### Detection of calcium influx with Fluo-4 AM in COS7 cells

COS7 cells were grown in 24-well plates and transfected with pcDNA3.1-ROP18-Flag individually or together with pcDNA3.1-P2X1-HA plasmids for 72 h. The cells were then harvested and washed twice with Hank’s balanced salt solution (HBSS; Thermo Fisher Scientific). Two hundred and fifty microliters of 5 μM Fluo-4 AM (an indicator of intracellular calcium ions; Dojindo Laboratories, Kumamoto, Japan) was added to the cells and incubated for 30 min at 37 °C. After being washed twice with HBSS, the cells were analyzed by FCM. Fluo-4 AM is virtually non-fluorescent, and acetoxymethyl (AM) ester moiety allows this dye to cross the cell membrane, whereupon endogenous esterases cleave the AM group to liberate the active dye and Ca^2+^ can bind to Fluo-4 intracellularly to emit green fluorescence. Time series reads were collected every 2 s for 100 s, and the mean value was calculated as the baseline read F0 (referred to as background). Following 60 µg/ml ATP stimulation, the green fluorescence intensity, reflecting the level of the free intracellular calcium ions in each group, was recorded for 500 s by FCM. The intracellular calcium level was quantified by the ratio of the strongest fluorescence signal to the basal signal (F/F0).

### Detection of mitochondrial membrane depolarization with JC-1 staining

SF268 cells were infected with RH or RH-Δ*rop18* tachyzoites (MOI = 13). Twelve hours later, 1 mg/ml ATP was added to the cells to induce apoptosis for 12 h. The cells were then digested with 0.25% trypsin and rinsed twice with PBS. Five hundred microliters of incubation buffer containing 1 μl of JC-1 was added to the cells and incubated for 15 min at 37 °C in 5% CO_2_. The cells were then visualized under a fluorescence microscope (Nikon) and subjected to FCM (BD Biosciences). Twenty views were obtained under GFP and RFP fluorescence filters, and the fluorescence intensities were determined using ImageJ software. This experiment was repeated four times for statistical analysis. For FCM, JC-1 was excited with a 488 nm argon laser, and JC-1 green and red fluorescence were recorded in the FL1 and FL2 channels. A minimum of 10,000 cells within the gated region were analyzed. The value was calculated by the relative intensities of green to red fluorescence. Staining with the lipophilic, cationic dye JC-1 can discriminate polarized and depolarized mitochondria, because in polarized mitochondria, the normal green fluorescent JC-1 dye forms red fluorescent aggregates in response to their higher membrane potential, while in depolarized mitochondria, the red fluorescent aggregates are converted to the monomeric form and exhibit green fluorescence [38].

### Separation of cytosolic and mitochondrial proteins in SF268 cells

Mitochondrial and cytosolic proteins were separated using a cell mitochondria isolation kit. SF268 cells were harvested and washed twice with cold PBS, and then incubated in 100 μl of ice-cold mitochondrial lysis buffer with gentle rotation at 4 °C for 15 min. The cell suspension was then transferred into a glass homogenizer and homogenized on ice for 30 strokes using a tight pestle. The homogenate was subjected to centrifugation at 600×*g* for 10 min at 4 °C to remove nuclei and unbroken cells. Then, the supernatant was collected and centrifuged again at 12,000×*g* for 10 min at 4 °C to obtain the cytosolic (supernatant) and mitochondrial (pellet) fractions. Cyt C, Bcl2 and Bax proteins in the cytosol and mitochondria were detected by western blotting. The intensity of Cyt C, Bcl2 and Bax bands with unsaturated exposure from four independent experiments were analyzed using ImageJ software, and the proportions of Cyt C, Bcl2 and Bax to the loading control Actin or COXIV were calculated.

### Western blotting

Protein samples were diluted in 6× SDS PAGE loading buffer and then boiled for 10 min. The boiled samples were loaded onto 15% SDS-polyacrylamide gels for electrophoresis and then transferred to a polyvinylidene fluoride (PVDF) membrane. After transfer, the PVDF membrane was blocked in PBS/5% bovine serum albumin (BSA)/0.05% Tween-20 at 37 °C for 2 h with gentle shaking, followed by probing with the primary antibody at 4 °C overnight. Then, the membrane was incubated with the secondary antibody at 37 °C for 2 h. The PVDF membranes were visualized by enhanced chemiluminescence (ECL) detection and photographed as recommended by the manufacturer.

### Statistical analysis

All experiments were repeated four times. The data were analyzed using GraphPad Prism 5 (San Diego, CA, USA). Statistical significance was determined using the Kruskal–Wallis H-test with Bonferroni correction, and *P < 0.05 and **P < 0.01 were considered to indicate a significant difference. The statistical comparisons presented in the figures are provided in Additional file 1: Table S1.

## Results

### Infection by three types of *T. gondii* strains (RH, ME49 and VEG) inhibited ATP-induced apoptosis of SF268, THP-1 and RAW264.7 cells

**Fig. 1:**
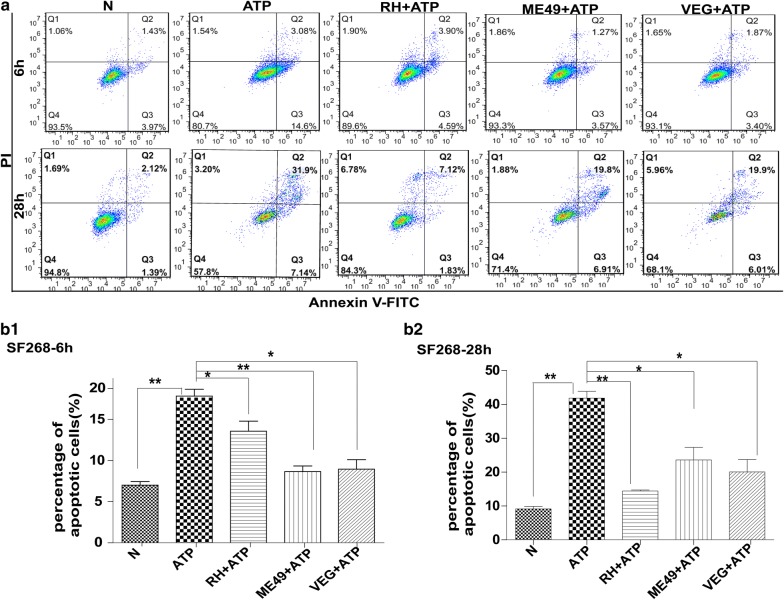
Effects of *T. gondii* infection on ATP-induced apoptosis of human SF268 neural cells. SF268 cells were infected with the RH, ME49 or VEG strain of *T. gondii* (MOI = 3) for 2 or 22 h, followed by ATP induction for 4 or 6 h. The cells were harvested at 6 or 28 h post-infection for apoptosis measurement by flow cytometry after annexin V-FITC/PI staining. Representative flow cytometry data are presented in panel **a** and quantified in panels **b1** and **b2**. The experiments were repeated four times. The values were analyzed using the Kruskal–Wallis H-test and Bonferroni correction (*P < 0.05, **P < 0.01)

**Fig. 2:**
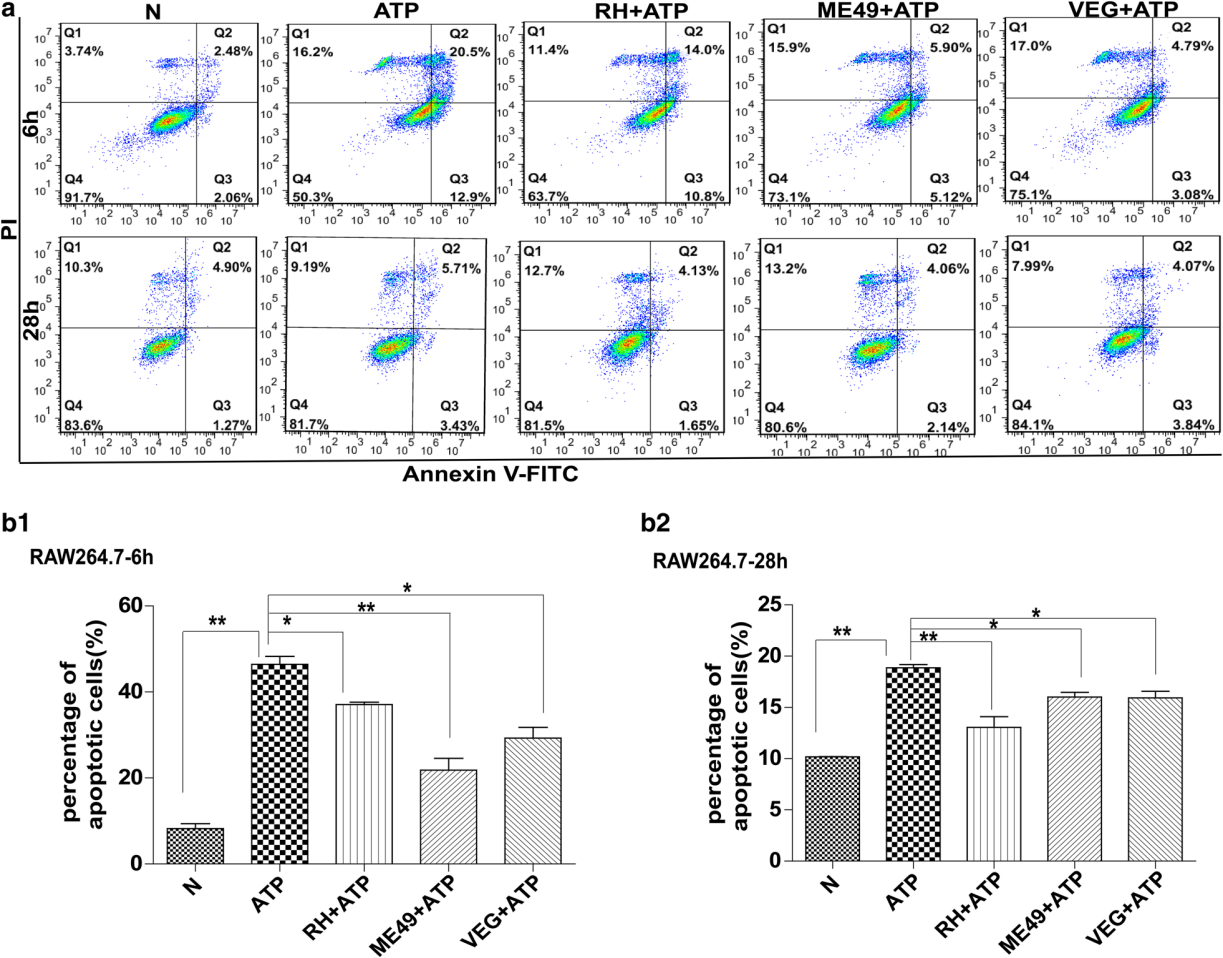
Effects of *T. gondii* infection on ATP-induced apoptosis of mouse RAW264.7 macrophage cells. RAW264.7 cells were infected with the RH, ME49 or VEG strain of *T. gondii* and followed by ATP induction 4 or 6 h. The cells were harvested at 6 or 28 h post-infection for apoptosis measurement by flow cytometry after annexin V-FITC/PI staining. Representative flow cytometry data are presented in panel **a** and quantified in panels **b1** and **b2**. The experiments were repeated four times. The values were analyzed using the Kruskal–Wallis H-test and Bonferroni correction (*P < 0.05, **P < 0.01)

**Fig. 3:**
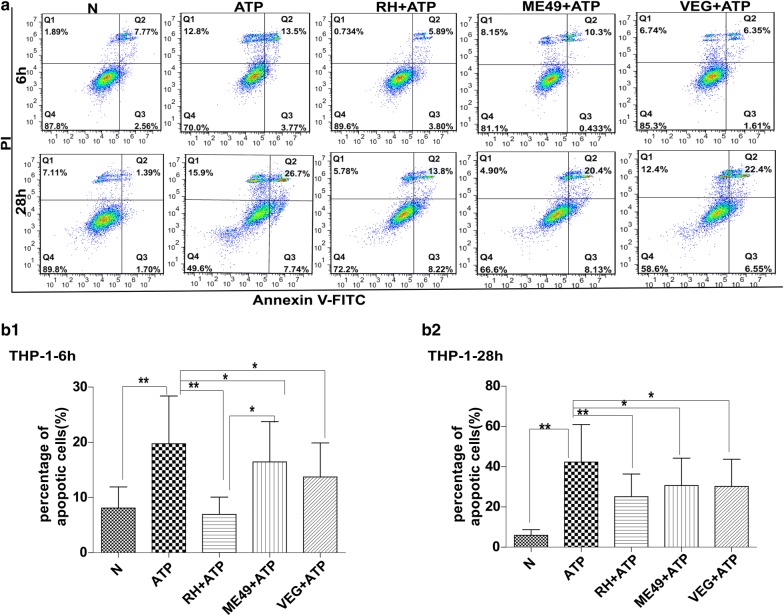
Effects of *T. gondii* infection on ATP-induced apoptosis of human THP-1 immune cells. THP-1 cells were infected with the RH, ME49 or VEG strain of *T. gondii* and followed by ATP induction 4 or 6 h. The cells were harvested at 6 or 28 h post-infection for apoptosis measurement by flow cytometry after annexin V-FITC/PI staining. Representative flow cytometry data are presented in panel **a** and quantified in panels **b1** and **b2**. The experiments were repeated four times. The values were analyzed using the Kruskal–Wallis H-test and Bonferroni correction (*P < 0.05, **P < 0.01)

Human SF268 neural cells were infected with *T. gondii* RH (type I), ME49 (type II) and VEG (type III), individually, for 2 or 22 h, and then were treated with ATP for 4 or 6 h to induce apoptosis. Our FCM results obtained with annexin V-FITC/PI staining indicated that there were significantly fewer apoptotic SF268 cells in any of the infected groups than that in the uninfected control, at both 6 and 28 h post-infection (Fig. 1a, b1, b2). Moreover, the TUNEL assay results also showed that *T. gondii* infection inhibited DNA fragmentation (Additional file 2: Figure S1). The same experiments were performed on the other two cell types, human THP-1 and mouse RAW264.7 monocyte/macrophage cells. The FCM results were similar to those observed with human SF268 glial cells (Fig. 2a, b1, b2 for RAW264.7 cells; Fig. 3a, b1, b2 for THP-1 cells). The TUNEL assay results obtained with these two types of cells also showed that the DNA fragmentation was less evident in any of the infected groups compared with their uninfected group (Additional file 3: Figure S2 for RAW264.7 cells; Additional file 4: Figure S3 for THP-1 cells). These results suggest that ATP-induced cell apoptosis was significantly inhibited by *T. gondii* infection, regardless of the tested strain types and host cell types.

### *Tg*ROP18 was a regulator for inhibition of ATP-induced apoptosis of SF268 cells

**Fig. 4:**
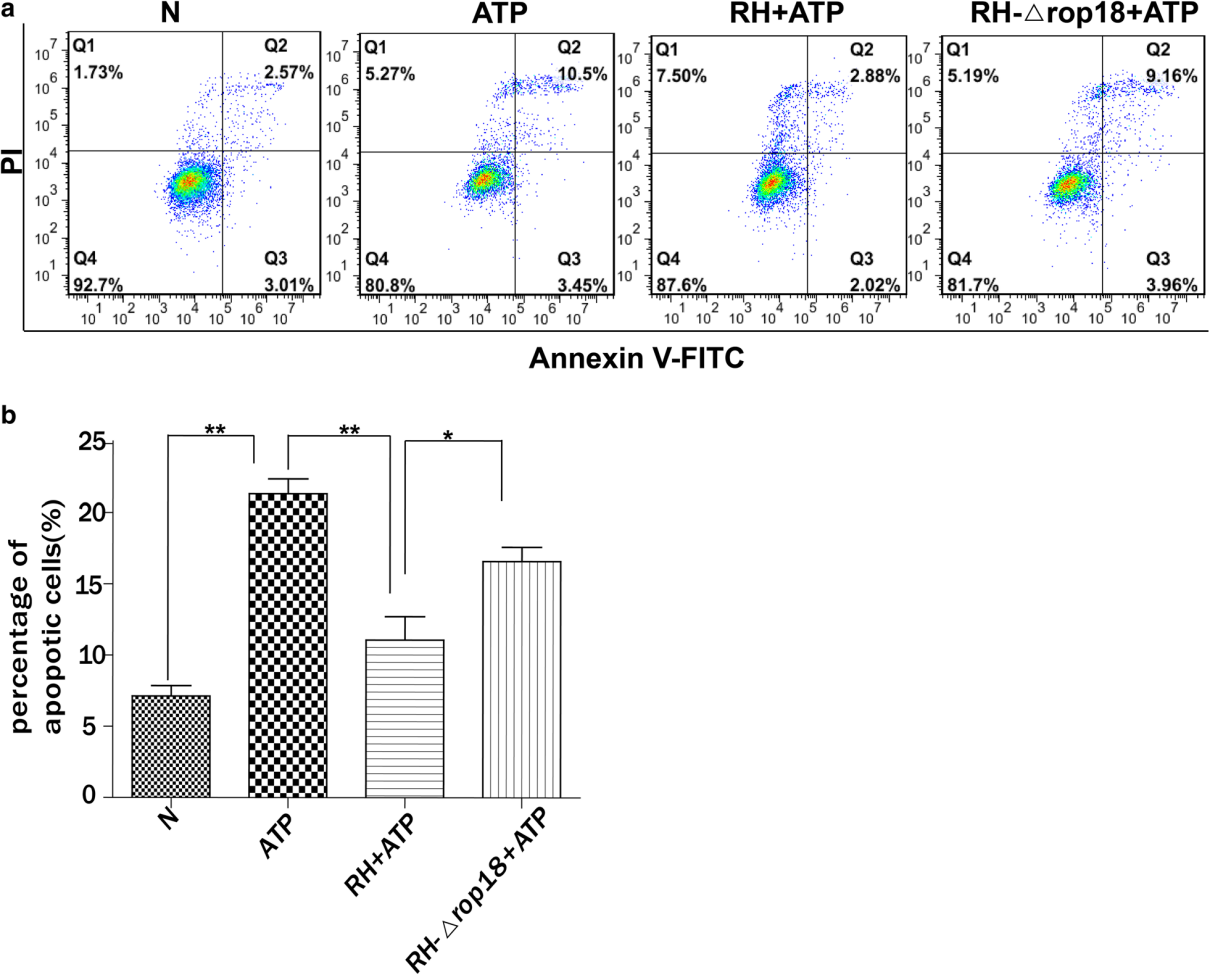
Inhibition of ATP-induced apoptosis of human SF268 neural cells by the *T. gondii* virulence factor ROP18. SF268 cells were infected with RH or RH-Δ*rop18* tachyzoites (MOI = 13) and followed by ATP induction at 12 h post-infection. The controls included SF268 cells without any treatment (N) and SF268 cells with ATP induction but without *T. gondii* infection (ATP). Apoptosis was measured by flow cytometry after annexin V-FITC/PI staining. Representative flow cytometry data are presented in panel **a** and quantified in panel **b**. The experiments were repeated four times. The values were analyzed using the Kruskal–Wallis H-test and Bonferroni correction (*P < 0.05, **P < 0.01)

Based on previous reports, *Tg*ROP18 of RH strain modulates host cell apoptosis, but the results are dependent on the type of cells infected by *T. gondii* [14, 15]. To evaluate whether apoptosis of SF268, THP-1 and RAW264.7 cells was influenced by *Tg*ROP18, the three cell types were infected with RH-Δ*rop18* or RH wild type tachyzoites at an MOI of 13 for 12 h, followed by ATP treatment for 12 h to induce apoptosis. Normal and ATP-treated cells were used as negative and positive controls, respectively. The FCM results showed that the apoptotic rate of the SF268 cells infected with RH strain was significantly lower than that of RH-Δ*rop18*-infected SF268 group (C2 = − 7, *df* = 3, *P* = 0.037) (Fig. 4a, b). However, this phenomenon was not observed in THP-1 and RAW264.7 cells, as both the RH and RH-Δ*rop18*-infected groups showed a lower percentage of apoptotic cells compared with the ATP treated group, and no significant difference was observed between the RH and RH-Δ*rop18*-infected groups (Additional file 5: Figure S4a, b). These results indicated that *Tg*ROP18 inhibited apoptosis of SF268 cells, but it played no observable role in modulating the apoptosis of THP-1 and RAW264.7 cells.

### *Tg*ROP18 targeted the host cell carboxyl-terminal region of P2X1

**Fig. 5:**
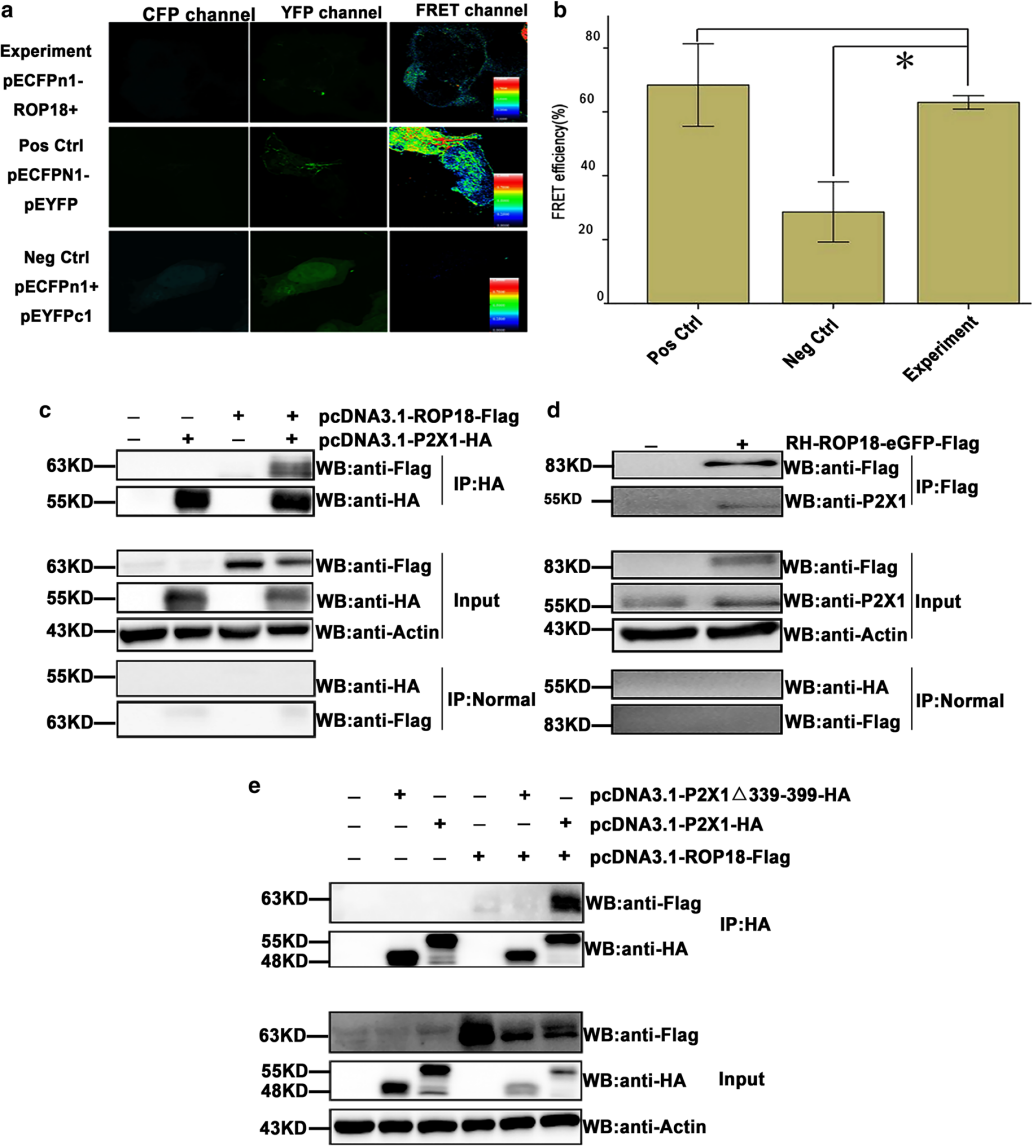
Identification of host P2X1 purinergic receptor as a *Tg*ROP18 target protein by FRET and co-IP. **a** Representative confocal FRET images. COS7 cells were cultured in plates the day before transfection. The experimental group was co-transfected with pECFP-N1-ROP18 and pEYFP-C1-P2X1, the negative control with pECFP-N1 and pEYFP-C1, and the positive control was transfected with pEYFP-CFP. The cells were fixed for confocal FRET measurement at 48 h post-infection. The vertical color bars represent FRET intensity, with red for a high FRET signal and blue for a low signal. **b** Quantitative analysis of FRET efficiency. **c** Co-IP analysis of COS7 cells transfected with pcDNA3.1-ROP18-3×Flag and/or pcDNA3.1-P2X1-HA. Lysates of the COS7 cells transiently transfected with the indicated plasmids or the control cells were immunoprecipitated with the anti-HA antibody and detected by western blotting with the indicated antibodies. **d** Co-IP analysis of SF268 cells infected with RH-ROP18-GFP-Flag. Lysates of the infected SF268 cells or the control cells were immunoprecipitated with anti-Flag antibody and detected by western blotting with the indicated antibodies. **e** Co-IP analysis of COS7 cells transfected with pcDNA3.1-ROP18-3×Flag and/or pcDNA3.1-P2X1-HA, pcDNA3.1-P2X1Δ339-399-HA. Lysates of the transfected COS7 cells or the control cells were immunoprecipitated with the anti-HA antibody and detected by western blotting with the indicated antibodies. The FRET efficiency was evaluated from 4 areas. The values were analyzed using the Kruskal–Wallis H-test and Bonferroni correction (*P < 0.05, **P < 0.01)

To understand the molecular mechanism by which *Tg*ROP18 inhibits cell apoptosis and identify its targets in host cells, we performed a genome-wide screening of human targets for *Tg*ROP18 of the RH strain with the bimolecular fluorescence complementation (BiFC) technique. The technique is based on two non-fluorescent fragments (YFP N-terminus and C-terminus) of the yellow fluorescent protein (YFP) that can reconstitute the fluorophore only when the two fragments are brought together by interactions between proteins that are covalently linked to each fragment [39]. P2X1 was identified as a putative interacting partner of *Tg*ROP18 [40]. To verify this interaction, we performed a FRET assay. For the FRET experiment, pECFP-ROP18 was a donor, and pEYFP-P2X1 an acceptor. If the interaction exists, the energy of the donor will transfer to the acceptor and they will reconstitute the functional fluorescent entity. As shown in Fig. 5a, the different color bands represent different ranges of FRET efficiencies: the dark blue represents the FRET efficiency of < 25%, the green for the FRET efficiency range of 25–50%, and the red for the FRET efficiency range of > 50%. The FRET efficiency of pECFP-ROP18 and pEYFP-P2X1 was significantly higher than pECFPn1-pEYFPc1 FRET efficiency. This further confirmed the interaction of *Tg*ROP18 with P2X1 in the cytosol of COS7 cells (Fig. 5a, b). The COS-7 cell line shows high efficiency of plasmid transfection and exogenous gene overexpression, and thus, it is very suitable for the overexpression of proteins for co-IP and FRET. SF268 is a human neural cell with P2X1 expression, which is suitable for co-IP of endogenous P2X1 and ROP18 after *T. gondii* infection. To further assess the specificity of this interaction, we overexpressed Flag-tagged *Tg*ROP18 together with HA-tagged P2X1 in COS7 cells, and the cell lysates were subjected to the immunoprecipitation assay using an anti-HA antibody. The results indicated that the overexpressed Flag-tagged *Tg*ROP18 was immunoprecipitated by the overexpressed HA-tagged P2X1 (Fig. 5c). The cell lysate of SF268 cells infected with RH-ROP18-GFP-Flag strain was immunoprecipitated with anti-Flag antibody, and the immunoblotting results indicated that the endogenous P2X1 from SF268 cells could be immunoprecipitated by *Tg*ROP18 tagged with Flag from the RH-ROP18-GFP-Flag strain (Fig. 5d).

Previous studies have shown that the C-terminus of the P2X1 receptor plays an important role in the regulation of its expression and gating activity [41]. To understand whether this domain was involved in binding to *Tg*ROP18, we constructed a pcDNA3.1-P2X1Δ339-399-HA plasmid with a deletion of the P2X1 C-terminus (amino acids 339–399) and co-transfected with pcDNA3.1-ROP18-3×Flag into COS7 cells. The results showed that Flag-tagged *Tg*ROP18 could not be immunoprecipitated by P2X1 carrying the C-terminal deletion when compared with the full-length P2X1, which was used as a positive control (Fig. 5e).

### *Tg*ROP18 decreased P2X1-mediated apoptosis

**Fig. 6:**
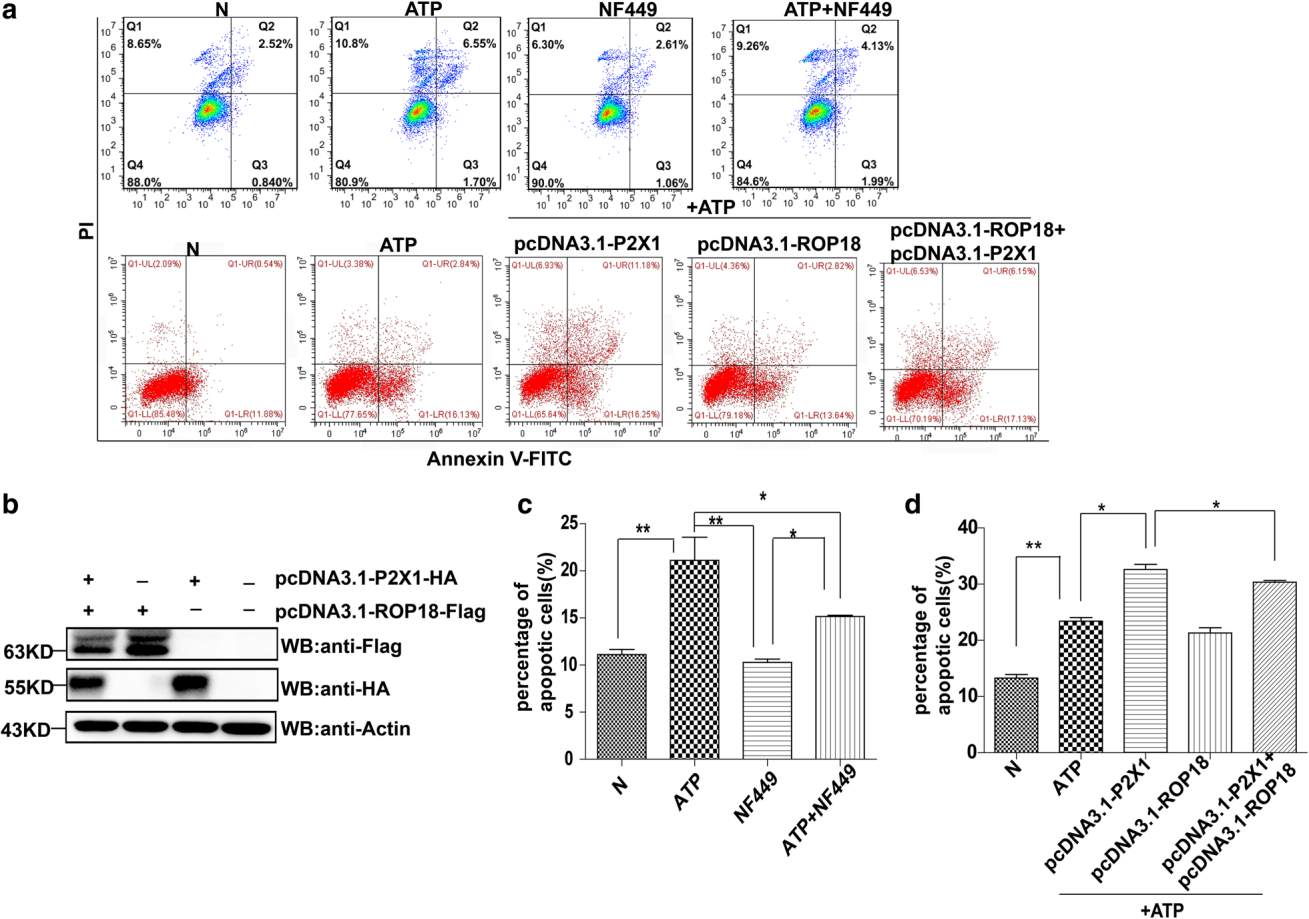
*Tg*ROP18 inhibition of host cell apoptosis promoted by P2X1. Four groups of SF268 cells were treated or not with ATP and/or NF449 as indicated. Five groups of COS7 cells were prepared. The N group is the normal COS7 cells. The other four groups were transfected with pcDNA3.1-ROP18 and/or pcDNA3.1-P2X1 and followed by treatment of ATP and/or NF449 as indicated. **a** Representative flow cytometry data. The apoptosis rate was measured after annexin V-FITC/PI staining. **b** Western blotting analysis of the expression of Flag-tagged ROP18 and HA-tagged P2X1 in COS7 cells. **c**, **d** Quantification of the flow cytometry data. The flow cytometry experiments were repeated four times. The values were analyzed using the Kruskal–Wallis H-test and Bonferroni correction (*P < 0.05, **P < 0.01)

When SF268 cells were pretreated with 4 µM NF449 (a specific inhibitor of P2X1 activity [32]) for 2 h before ATP treatment, the apoptosis rate was significantly lower in the NF449 + ATP-treated group than the ATP-treated group (C2 = 7.735, *df* = 3, *P* = 0.025), indicating that P2X1 was indeed involved in ATP-induced apoptosis (Fig. 6a, c). In contrast, we found that the apoptosis index in the NF449 + ATP treated group was significantly higher than in the NF449-treated group (C2 = 6.875, *df* = 3, *P* = 0.04), although both of their apoptosis indexes were significantly lower than the ATP-treated group (Fig. 6a, c). These results suggested that P2X1 inhibitor NF449 could not completely inhibit the apoptosis induced by ATP, and some proapoptotic proteins other than P2X1 likely regulated SF268 apoptosis induced by ATP.

To further identify whether *Tg*ROP18 inhibited host cell apoptosis through binding to the proapoptotic protein P2X1 of the host cell, untransfected COS7 cells and COS7 cells transfected with pcDNA3.1-*Tg*ROP18 and pcDNA3.1-P2X1, individually or together for 48 h, were stimulated with ATP for an additional 24 h. Normal cells (N, untransfected and untreated with ATP) served as the control. The expression of these two proteins was verified by western blotting (Fig. 6b). Cell apoptosis was assessed by FCM following annexin V-FITC/PI staining. The results showed that COS7 cells overexpressing P2X1 had a significantly higher apoptosis index compared with untransfected cells and cells with overexpression of *Tg*ROP18, whereas co-expression of *Tg*ROP18 with P2X1 in COS7 cells significantly decreased ATP-induced apoptosis compared with cells expressing only P2X1 (C2 = 8.375, *df* = 4, *P* = 0.045) (Fig. 6d).

### *Tg*ROP18 decreased host cell apoptosis through inhibition of P2X1-mediated Ca^2+^ influx, but not through P2X1 degradation

**Fig. 7:**
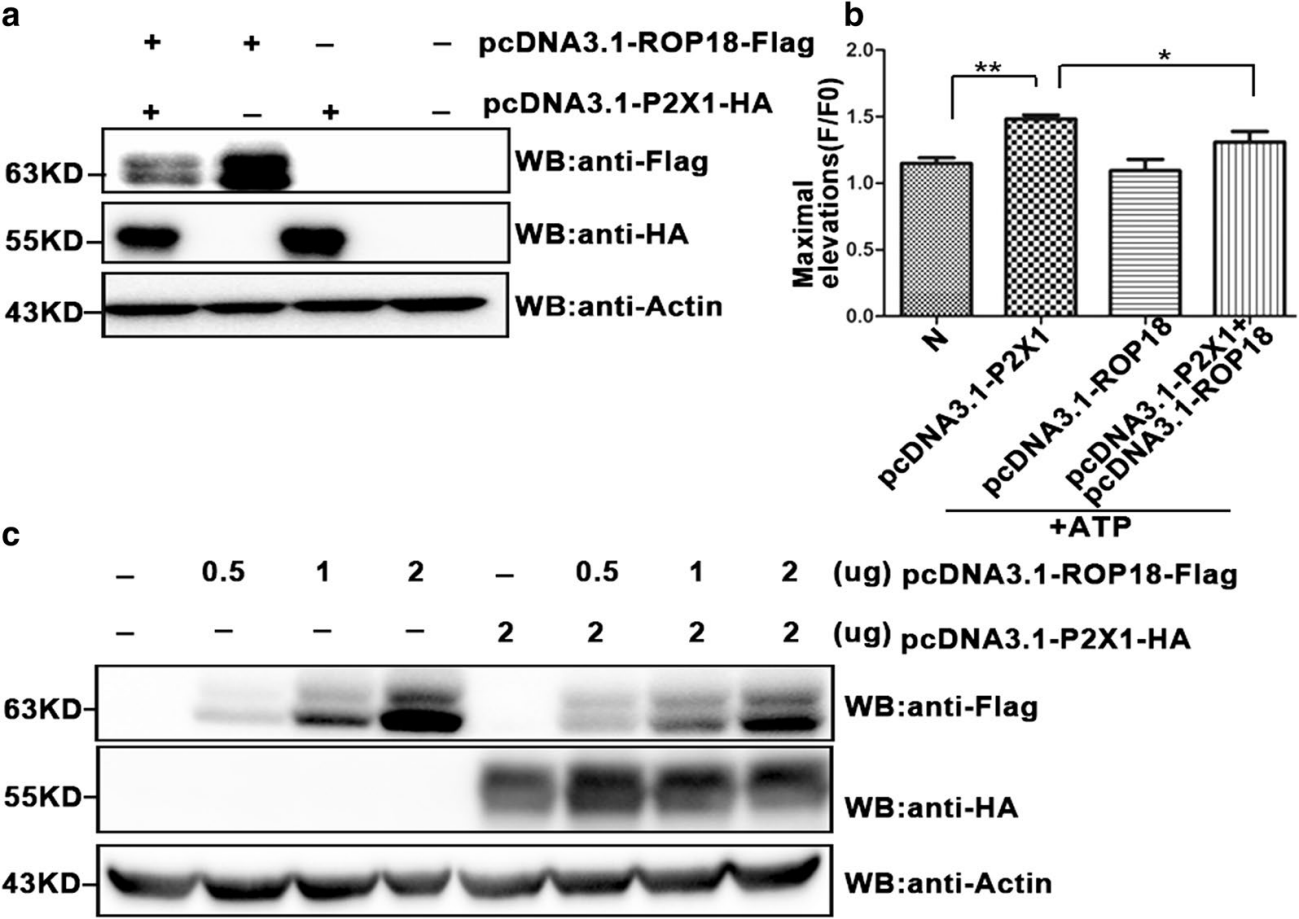
*Tg*ROP18 inhibition of P2X1-mediated calcium influx. **a** Expression of Flag-tagged ROP18 and HA-tagged P2X1. COS7 cells were transfected with pcDNA3.1-P2X1-HA and/or pcDNA3.1-ROP18-3×Flag. Production of the ROP18 and P2X1 proteins was determined by western blotting. **b** Calcium influx measurement. The transfected cells in A were stimulated with ATP, and Ca^2+^ influx in each group was analyzed using flow cytometry. The maximum elevation (F/F0) of the intracellular Ca^2+^ were analyzed using the Kruskal–Wallis H-test and Bonferroni correction (*P < 0.05). **c** P2X1 protein integrity. COS7 cells were transfected with a stable amount (2.0 µg) of pcDNA3.1-P2X1-HA and sequentially co-transfected with 0, 0.5, 1.0 and 2.0 µg of pcDNA3.1-ROP18-3×Flag. The integrity of the proteins was analyzed by western blotting

*Tg*ROP18 and P2X1 were overexpressed in COS7 cells for 72 h, and the intracellular Ca^2+^ concentration was measured for 600 s following the addition of 60 µg/ml ATP into the culture medium. We found that P2X1 increased Ca^2+^ influx after ATP stimulation, and this process could be inhibited by co-expression of *Tg*ROP18 in the COS7 cells (C2 = 7, *df* = 3, *P* = 0.036) (Fig. 7a, b). However, the P2X1-mediated Ca^2+^ increase was not completely inhibited by *Tg*ROP18, this indicated that besides P2X1, some other proapoptotic proteins are also involved in regulation of COS7 cells apoptosis. Considering that *Tg*ROP18 can phosphorylate host cell proteins ATF6β and P65 for degradation to suppress the host innate immune response [12, 13], we next examined whether ROP18 caused P2X1 degradation. COS7 cells were co-transfected with pcDNA3.1-ROP18-Flag and/or pcDNA3.1-P2X1-HA expression vectors for 48 h. The western blot results showed that ROP18 expression did not affect the protein level of P2X1 (Fig. 7c). All these findings revealed that ROP18 inhibited P2X1-mediated Ca^2+^ influx without causing P2X1 degradation, despite their interaction.

### *Tg*ROP18 inhibited mitochondrial depolarization and Cyt C translocation from mitochondria to the cytosol in SF268 cells

**Fig. 8:**
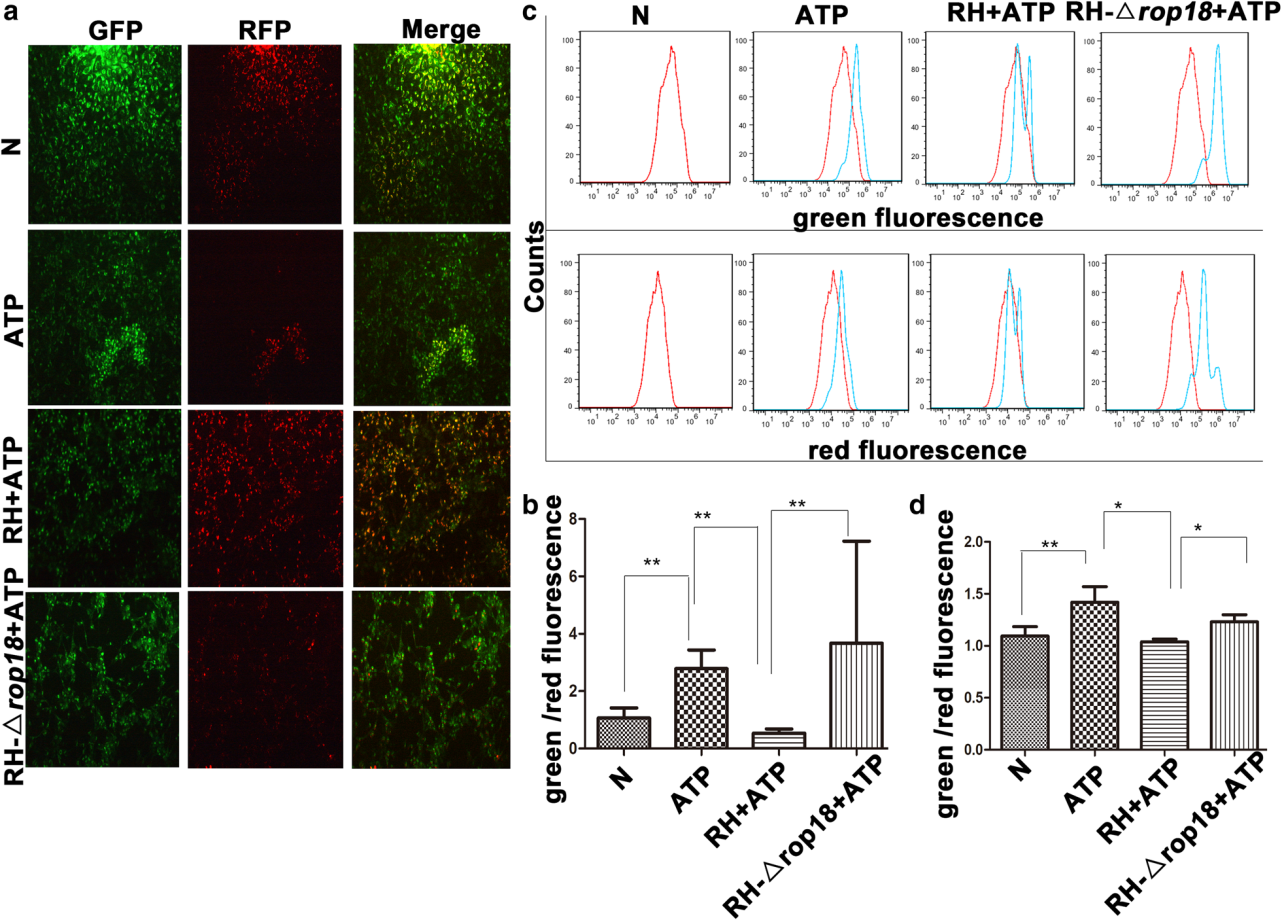
*Tg*ROP18 inhibition of ATP-induced mitochondrial depolarization in SF268 cells. SF268 cells were infected with RH or RH-Δ*rop18* tachyzoites at MOI of 13 and followed by ATP induction of apoptosis. **a** Visualization of mitochondrial membrane depolarization by JC-1 staining and fluorescence microscopy (10×). **c** Flow cytometric analysis of mitochondrial membrane depolarization. **b**, **d** Quantification of mitochondrial membrane depolarization. The percentages of green to red fluorescence were determined using a fluorescence microscope and FCM for each group of cells. The experiments were repeated four times. The values were analyzed using the Kruskal–Wallis H-test and Bonferroni correction (**P < 0.01, *P < 0.05)

Previous studies have shown that RH regulates apoptosis of host cells mainly through the mitochondrial pathway [42]. To identify whether this pathway was modulated by *Tg*ROP18 during *T. gondii* infection, we first measured the mitochondrial membrane potential after SF268 cells were infected by RH or RH-Δ*rop18* strain for 12 h followed by ATP treatment for 12 h. When the mitochondrial membrane is normal, the cationic dye JC-1 forms red fluorescent aggregates accumulated in mitochondria in response to their higher membrane potential, while in a broken mitochondrial membrane, the red fluorescent aggregates are converted to the monomeric form and exhibit green fluorescence accumulated in the cytosol. The intensity ratio of green to red fluorescence reflects the extent of mitochondrial membrane depolarization. Both fluorescence microscopy observation (Fig. 8a, b) and FCM analysis (Fig. 8c, d) showed that the intensity ratio of green to red fluorescence decreased in the RH + ATP-treated group compared with the ATP-treated group (C2 = 26, *df* = 3, *P* = 0.000; C2 = 8.25, *df* = 3, *P* = 0.014), indicating that infection of the RH strain inhibited mitochondrial membrane depolarization induced by ATP. Moreover, the intensity ratio of green to red fluorescence also decreased in the RH + ATP treatment group compared with the RH-Δ*rop18* + ATP treatment group (C2 = − 22.95, *df* = 3, *P* = 0.000; C2 = − 6.75, *df* = 3, *P* = 0.045), implying that depolarization of the mitochondrial membrane was inhibited by *Tg*ROP18. Therefore, an increase in green/red fluorescence ratio means depolarization of the mitochondrial membrane and this phenomenon can be changed by the expression of *Tg*ROP18.

**Fig. 9:**
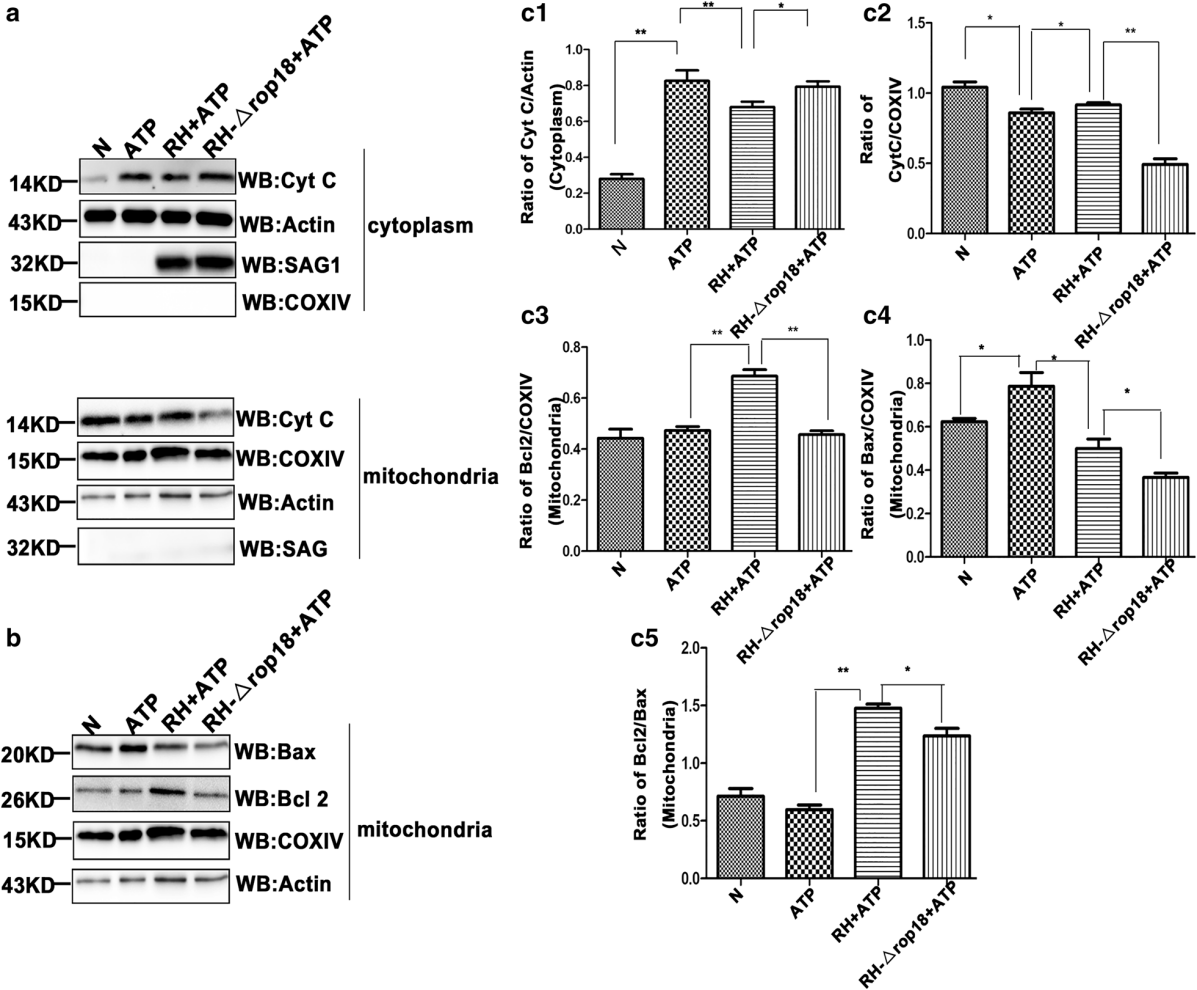
*Tg*ROP18 inhibition of Cyt C release from mitochondria to the cytosol in ATP-treated SF268 cells. SF268 cells were harvested and lysed, and the mitochondria and cytosol were fractionated. **a**, **b** Western blot analysis of protein distributions in the mitochondria and cytosol. The data represent one of the four repeated experiments. **c** Densitometric analysis of the western blotting images. In four repeated experiments, the ratios of Cyt C/actin, Cyt C/COXIV, Bcl2/COXIV, Bax/COXIV and Bcl2/Bax were calculated independently. Statistical analysis was performed using the Kruskal–Wallis H-test and Bonferroni correction (**P < 0.01, *P < 0.05) (**c1**, cytosolic fraction; **c2**–**c5** mitochondrial fraction)

The mitochondrial protein Cyt C plays an important role in initiating the intrinsic apoptotic pathway. The anti-and the pro-apoptotic family of proteins is tightly associated with the integrity of the mitochondrial outer membrane and release of Cyt C from mitochondria to the cytosol. To evaluate the apoptotic consequences mediated by *Tg*ROP18, we next measured the release of Cyt C from mitochondria to the cytosol and the translocation of Bax (a pro-apoptotic Bcl2 family protein) and Bcl2 (an anti-apoptotic Bcl2 family protein) from the cytosol to mitochondria in each group. The western blot results revealed a relatively higher level of Cyt C in the mitochondrial fraction, but a lower level of Cyt C was detected in the cytosolic fraction in the RH + ATP treatment group compared with the RH-Δ*rop18* + ATP treatment group (C2 = − 6.75, *df* = 3, *P* = 0.044; C2 = 12, *df* = 3, *P* = 0.000) (Fig. 9a, c1, c2). These findings indicated that *Tg*ROP18 inhibited cyt C translocation from the mitochondria to the cytosol, and promoted the translocation of anti-apoptotic protein Bcl2, proapoptotic protein Bax from the cytosol to mitochondria.

Moreover, an increased level of anti-apoptotic protein Bcl2 (C2 = 9.5, *df* = 3, *P* = 0.005) and decreased level of proapoptotic protein Bax (C2 = − 11.75, *df* = 3, *P* = 0.000) relative to COXIV was found in the mitochondrial fraction of the RH + ATP treatment group compared with the ATP-treated group (Fig. 9b, c3, c4), indicating that RH infection promoted the translocation of Bcl2 and inhibited the translocation of Bax from the cytosol to mitochondria in SF268 cells. Conversely, the protein level of mitochondrial Bcl2 relative to COXIV was markedly higher in the RH + ATP than the RH-Δ*rop18* + ATP treatment group (C2 = 9.375, *df* = 3, *P* = 0.005) (Fig. 9b, c3). As a pro-apoptotic protein, translocation of Bax is supposedly inhibited by *Tg*ROP18; however, we found that *Tg*ROP18 significantly increased the relative Bax level in the mitochondrial fraction by comparing the RH + ATP and RH-Δ*rop1*8 + ATP treatment groups (C2 = 7.75, *df* = 3, *P* = 0.021) (Fig. 9b, c4). These results indicated that *Tg*ROP18 increased the relative level of the anti-apoptotic protein Bcl2 as well as the proapoptotic protein Bax in the mitochondrial fraction. Our analysis further showed that the Bcl2/Bax ratio at the protein level in the mitochondrial fraction was significantly higher in the RH + ATP than the RH-Δ*rop18* + ATP group (C2 = 8, *df* = 3, *P* = 0.017) (Fig. 9b, c5). These results suggested that *Tg*ROP18 protected mitochondrial depolarization and translocation of Cyt C from mitochondria to the cytosol. COX IV and actin were detected as controls for the mitochondrial and cytosolic fraction, respectively.

### *Tg*ROP18 inhibited ATP-triggered caspase activation

**Fig. 10:**
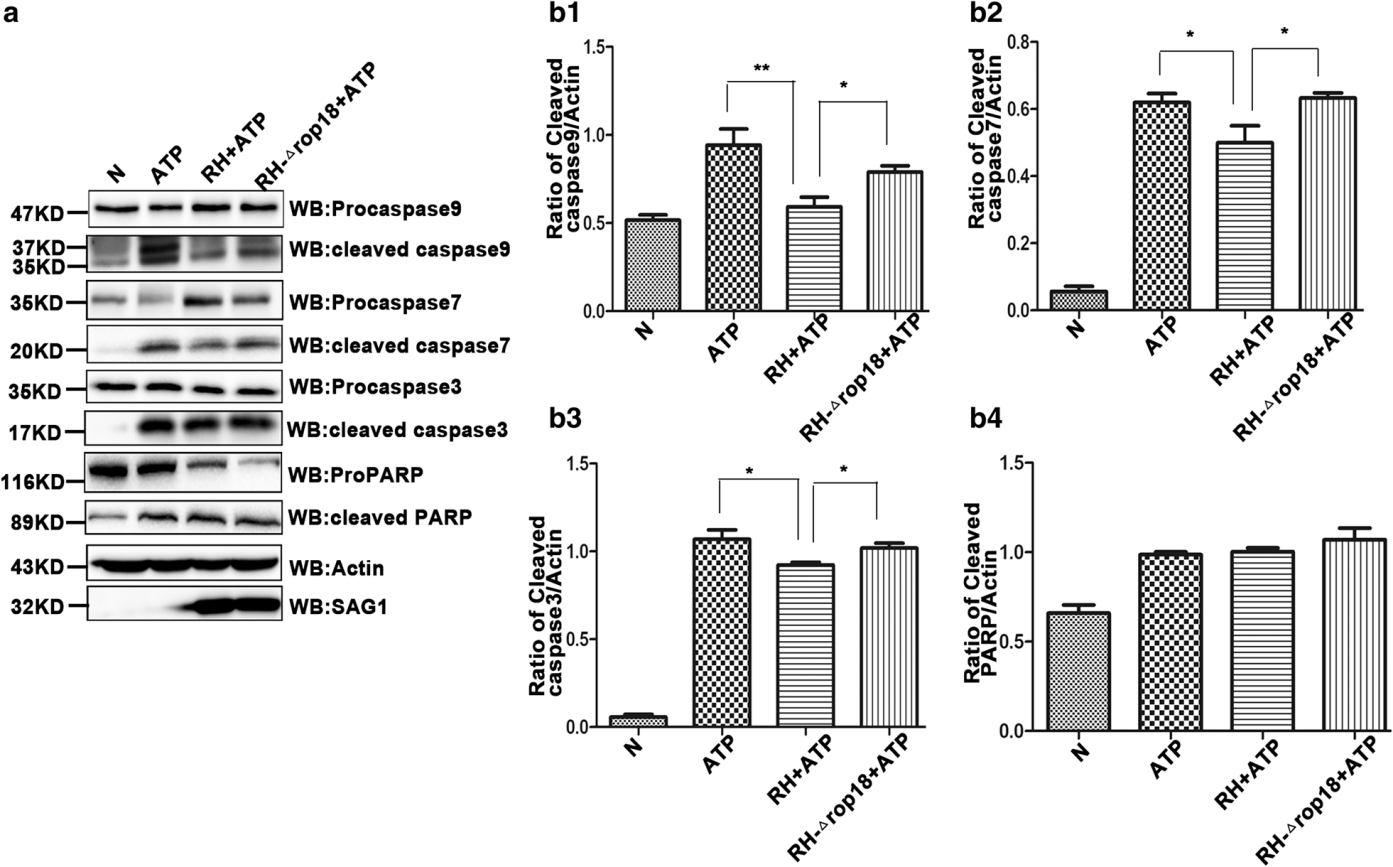
*Tg*ROP18 inhibition of procaspase-3, procaspase-7 and procaspase-9 cleavage to form active caspases in ATP-treated SF268 cells. **a** Western blot analysis of procaspase cleavage. SF268 cells were prepared as described in Fig. 6. Cell lysates were used for western blot analysis. The antibodies used could recognize both the full-length and cleaved forms of their protein antigens. The molecular weights of the full-length and cleaved forms of the protein antigens are as follows. caspase-9: 47, 37, 35 kD; caspase-7: 35, 20 kD; caspase-3: 35, 17 kD; PARP: 116, 89 kD. Actin was used as a control, and SAG1 was used to evaluate the number of *T. gondii* tachyzoites, ensuring consistent numbers of host cells and tachyzoites in comparison groups. **b** Densitometric analysis of the western blotting images. Quantification was performed using ImageJ software, and the experiments were repeated four times for statistical analysis. The comparison the ratios of cleaved caspase-9/actin (**b1**), cleaved caspase-7/actin (**b2**), cleaved caspase-3/actin (**b3**), and cleaved PARP/actin (**b4**) between group was performed using the Kruskal–Wallis H-test and Bonferroni correction (**P < 0.01, *P < 0.05)

The mitochondrial apoptotic pathway depends mostly on caspase-9, which in turn activates executioner caspase-3 and caspase-7 [43, 44]. Our immunoblotting results indicated that in ATP-treated SF268 cells, inactive full-length caspase-9 was cleaved into active p35 and p37 fragments, inactive full-length caspase-7 was cleaved into active p20 fragments, and inactive full-length caspase-3 was split into p17 fragments. In SF268 cells treated with RH + ATP but not RH-Δ*rop18* + ATP, processing of all these three procaspases was significantly inhibited. In contrast, no significant difference was detected in the levels of cleaved PARP (poly ADP-ribose polymerase) between the ATP-treated and RH + ATP groups (Fig. 10). The results suggest that *Tg*ROP18 inhibits activation of these procaspases in SF268 cells.

## Discussion

Based on previously reported findings, we observed variability in apoptosis regulated by type Ӏ strain RH and type II strain (NTE or ME49) in immune cells or neural cells (Additional file 6: Table S2), and found that *T. gondii* infection can both inhibit and induce host cell apoptosis in different settings. Whether these opposite effects of *T. gondii* infection are due to variations in strain virulence and/or host cell types is not clear. To address this issue, we carried out a three by three combination study under the same conditions (Figs. 1, 2, 3). Our study employed three *T. gondii* strains with different degrees of virulence, the virulent RH-type I strain and the non-virulent ME49-type II and VEG-type III strains, and three host cell lines, human SF268 neural cells, human RAW264.7 and mouse THP-1 immune cells, representing two most important hosts and two most frequently infected cell types. The results indicate that *T. gondii* infection inhibits ATP-induced apoptosis, regardless of strain virulence and host cell lines tested. This is consistent with most reports on the effects of *T. gondii* infection on apoptosis of a variety of host cells [7, 8, 42, 45–49], and it is to *T. gondii*’s advantage to inhibit host cell apoptosis and keep the host cells alive [50–52].

It is thought that the induction of host immune cell apoptosis may restrict host immune response and allow *T. gondii* to establish infection [49, 53]. *Toxoplasma gondii* infection has also been found to induce the apoptosis of mouse N2A neural cells [14] and JEG-3 choriocarcinoma cells [54]. However, it is not obvious that the induction of the apoptosis of these cells has any advantage to *T. gondii*’s survival.

The next questions we addressed are which virulence factor of *T. gondii* is involved in the inhibition of ATP-induced apoptosis and which host protein is targeted by the virulence factor of the pathogen. We investigated *Tg*ROP18, as it is a major virulence factor of *T. gondii*, and found that *Tg*ROP18 significantly inhibited the ATP-induced apoptosis of SF268 cells (Fig. 4) but not THP-1 and RAW264.7 cells (Additional file 5: Figure S4). The P2X1 purinergic receptor was found to form a complex with *Tg*ROP18 (Fig. 5). *Tg*ROP18 suppressed host cell apoptosis through inhibition of P2X1-mediated Ca^2+^ influx, but not through P2X1 degradation (Figs. 6, 7).

Then, we addressed the question of why *Tg*ROP18 inhibited the apoptosis of SF268 cells but not that of THP-1 and RAW264.7 cells. We found that P2X1 was expressed in SF268 cells but not in THP-1 and RAW264.7 cells (Additional file 7: Figure S5), and *Tg*ROP18 can bind with P2X1 to inhibit the ATP-induced cell apoptosis (Fig. 5). Owing to lack of P2X1 expression in COS7 cells, the overexpression of ROP18 in COS7 cells did not influence the cell apoptosis (Fig. 6d). Although P2X1 expression was not detected in RAW264.7 and THP-1 cells, the apoptosis of RAW264.7 can be induced by activation of the protein kinase extracellular regulated protein kinase (ERK) and mitogen-activated protein kinase (MAPK) pathway regulated by extracellular signals through P2X4 as well as P2X7 receptors [55]; and THP-1 cell apoptosis can be promoted by the increase in intracellular calcium through the P2X7 receptor [56]. Therefore, it seemed that ROP18 could only target P2X1, but not P2X4 or P2X7, to regulate host cell apoptosis. On the other hand, we also found that the P2X1 inhibitor NF449 failed to completely inhibit ATP-induced apoptosis. Thus, some pro-apoptotic receptors besides P2X1 in SF268 cells may respond to ATP stimulation to regulate the cell apoptosis.

Apoptosis can be induced by two signal transduction pathways, one termed the intrinsic pathway or the mitochondrial pathway and the other the extrinsic pathway [16, 57]. Consequently, we investigated the mechanism by which *Tg*ROP18 inhibited the ATP-induced apoptosis. We found that *Tg*ROP18 inhibited mitochondrial calcium influx (Fig. 7) and depolarization (Fig. 8), the release of Cyt C from mitochondria to the cytosol (Fig. 9) and caspase maturation (Fig. 10). The first two represent the fundamental function of the P2X1 purinergic receptor, and the latter two are the key steps in the mitochondrial apoptotic pathway. The initial steps of the mitochondrial apoptotic pathway are mediated by the Bcl-2 family of proteins, including anti-apoptotic Bcl-2 proteins and proapoptotic proteins Bax and Bak. In normal cells, Bcl2 and Bax are located predominantly in the cytosol [58], but under apoptotic conditions, they accumulate at the mitochondrial outer membrane [59]. Cyt C is a small hemeprotein that is found loosely associated with the mitochondrial inner membrane (MIM), where it plays a crucial role in transferring electrons [33]. Apoptotic signals such as DNA damage can increase permeabilization of the MIM, and Cyt C will be released from the MIM medium to the cytosol [60]. Whether Cyt C is released into the cytosol is partly dependent on the ratio of Bcl2/Bax [60]. Our analysis demonstrated that the Bcl2/Bax ratio at the protein level was significantly elevated by *Tg*ROP18 in the mitochondrial fraction (Fig. 9), indicating that the release of Cyt C from mitochondria to the cytosol is inhibited by *Tg*ROP18. The results showed that *Tg*ROP18 inhibits ATP-induced apoptosis by interfering with the mitochondrial apoptotic pathway.

Although *Tg*ROP18 expression in type III strains can barely be detected, we found in this study that type III strain VEG could also inhibit host cell apoptosis. The other *T. gondii* factors may function in the inhibition of host cell apoptosis independently of *Tg*ROP18. It has been reported that *Tg*ROP38 is normally undetectable in the virulent RH strain [61]; however, it is abundant in the avirulent VEG strain at the transcriptional level, and expression of *Tg*ROP38 exerts a potent effect on the regulation of cell proliferation and apoptosis through the MAPK pathway [62]. Several other *T. gondii* virulence factors have been found to modulate host cell apoptosis, including *T. gondii* ROP38 (another rhoptry protein kinase) [62], type 2C protein phosphatase (PP2C) [63] and dense granule protein GRA15 [54]. Whether these and other *T. gondii* virulence factors also play a role in modulating ATP-induced host cell apoptosis needs to be further investigated.

## Conclusions

The present findings showed that *T. gondii* infection inhibits ATP-induced host cell apoptosis, regardless of strain virulence and host cell lines. *Toxoplasma gondii* virulence factor *Tg*ROP18, which targeted the C-terminus of P2X1, inhibited apoptosis mediated by P2X1. When apoptosis was induced by ATP, the P2X1 mediated Ca^2+^ influx was inhibited by the presence of *Tg*ROP18 in COS7 cells overexpressing both P2X1 and *Tg*ROP18. Furthermore, *Tg*ROP18 inhibited depolarization of the mitochondrial membrane, Cyt C translocation from the mitochondria to the cytoplasm, and ATP-triggered caspase 9, 7 and 3 activation in SF268 cells. Taken together, these findings support the conclusion that *Tg*ROP18 inhibits ATP-induced apoptosis by interfering with P2X1 function and the mitochondrial apoptotic pathway.

## Additional files

**Additional file 1: Table S1.** Summarized information of statistical comparisons presented in the figures.

**Additional file 2: Figure S1.** TUNEL assay of ATP-induced apoptosis of infected SF268 cells. SF268 cells were infected with RH, ME49 or VEG strain and treated with ATP in the same way as described in Fig. 1. **a1**, **a2** Analysis of apoptosis by fluorescence microscopy following TUNEL assay. TUNEL-positive cells were visualized as indicated by green fluorescence staining and DAPI (blue) staining to discern all cell nuclei. The percentage (%) of TUNEL-positive cells was determined (number of TUNEL-positive cells/total number of cells × 100). **b1**, **b2** Quantification of the data in panels **a1** and **a2**. The experiments were repeated four times. The values were analyzed using the Kruskal–Wallis H-test and Bonferroni correction (**P < 0.01).

**Additional file 3: Figure S2.** TUNEL assay of ATP-induced apoptosis of infected RAW264.7 cells. RAW264.7 cells were infected with the RH, ME49 or VEG strain and treated with ATP in the same way as described in Fig. 1. Data analysis and presentation were performed in the same way for the corresponding panels of Figure S1. The experiments were repeated four times. The values were analyzed using the Kruskal–Wallis H-test and Bonferroni correction (**P < 0.01).

**Additional file 4: Figure S3.** TUNEL assay of ATP-induced apoptosis of infected THP-1 cells. THP-1 cells were infected with the RH, ME49 or VEG strain and treated with ATP in the same way as described in Fig. 1. Data analysis and presentation were performed in the same way for the corresponding panels of Figure S1. The experiments were repeated four times. The values were analyzed using the Kruskal–Wallis H-test and Bonferroni correction (**P < 0.01).

**Additional file 5: Figure S4.** Effect of *T. gondii* virulence factor ROP18 on ATP-induced apoptosis of RAW264.7 and THP-1 cells. RAW264.7 and THP-1 cells were infected with RH or RH-Δ*rop18* tachyzoites (MOI = 13) or left uninfected to serve as the normal control (N) or positive control (ATP treatment). At 12 h post-infection, 1 mg/ml ATP was added to the cells for an additional 12 h, except in the normal control group. **a** Representative flow cytometry data. **b** Quantification of the flow cytometry data. The percentages of apoptotic cells were separately determined for each group of cells. The experiments were repeated four times for Kruskal–Wallis H-test statistical analysis (*P < 0.05, **P < 0.01).

**Additional file 6: Table S2.** Summarized information for *Toxoplasma gondii* modulation of immune and neural cell apoptosis.

**Additional file 7: Figure S5.** Western blot analysis of P2X1 in SF268, RAW264.7, HFF and THP-1 cells. RAW264.7, HFF, THP-1 and SF268 cells were grown in a T25 flask to 100% confluence and then harvested and lysed. Total proteins for each sample were subjected to SDS-PAGE and western blotting analysis with P2X1 antibody.

## Data Availability

The datasets supporting the findings of this article are included within the article and its additional files.

## Abbreviations

P2X1: purinergic receptor
*Tg*ROP18: *Toxoplasma gondii* rhoptry protein 18
ATP: adenosine triphosphate
Cyt C: cytochrome C
COXIV: cytochrome C oxidase IV
PARP: poly ADP-ribosepolymerases
MIM: mitochondria inner membrane
