# Supplementary material for: Toxoplasma gondii ROP18 inhibits human glioblastoma cell apoptosis through a mitochondrial pathway by targeting host cell P2X1
Source: Parasit Vectors. 2019 Jun 4;12:284. doi: 10.1186/s13071-019-3529-1 (PMC6547611; doi:10.1186/s13071-019-3529-1)
Supplement: Supplementary file 1 — Additional file 1: Table S1. Summarized information of statistical comparisons presented in the figures. [file 13071_2019_3529_MOESM1_ESM.docx]

**Additional file 1: Table S1.** Statistical comparisons presented in the figures

**Figure1-b1 C2=14.729, df=4, p=0.001**

| Comparing groups | C2 value | P value corrected by Bonferroni |
| --- | --- | --- |
| N vs ATP | -14.5 | 0.001 |
| ATP vs RH+ATP | 8 | 0.032 |
| ATP vs ME49+ATP | 11 | 0.009 |
| ATP vs VEG+ATP | 9.75 | 0.02 |
| RH+ATP vs ME49+ATP | 6.25 | 0.135 |
| RH+ATP vs VEG+ATP | 5 | 0.232 |
| ME49+ATP vs VEG+ATP | -1.25 | 0.765 |

**Figure 1-b2. C2=18.286, df=4, p=0.001**

| Comparing groups | C2 value | P value corrected by Bonferroni |
| --- | --- | --- |
| N vs ATP | -16 | 0.000 |
| ATP vs RH+ATP | 12 | 0.004 |
| ATP vs ME49+ATP | 10 | 0.01 |
| ATP vs VEG+ATP | 9.25 | 0.03 |
| RH+ATP vs ME49+ATP | -8 | 0.056 |
| RH+ATP vs VEG+ATP | -4 | 0.339 |
| ME49+ATP vs VEG+ATP | 4 | 0.339 |

**Figure 2-b1. C2=17.412, df=4, p=0.002**

| Comparing groups | C2 value | P value corrected by Bonferroni |
| --- | --- | --- |
| N vs ATP | -16 | 0.000 |
| ATP vs RH+ATP | 8.25 | 0.048 |
| ATP vs ME49+ATP | 11.75 | 0.005 |
| ATP vs VEG+ATP | 9.25 | 0.02 |
| RH+ATP vs ME49+ATP | 7.75 | 0.064 |
| RH+ATP vs VEG+ATP | 4.25 | 0.309 |
| ME49+ATP vs VEG+ATP | -3.5 | 0.402 |

**Figure 2-b2 C2=17.412, df=4, p=0.002**

| Comparing groups | C2 value | P value corrected by Bonferroni |
| --- | --- | --- |
| N vs ATP | -16 | 0.000 |
| ATP vs RH+ATP | 12 | 0.004 |
| ATP vs ME49+ATP | 9 | 0.03 |
| ATP vs VEG+ATP | 9.05 | 0.025 |
| RH+ATP vs ME49+ATP | -5.75 | 0.169 |
| RH+ATP vs VEG+ATP | -6.25 | 0.135 |
| ME49+ATP vs VEG+ATP | 0.5 | 0. 905 |

| Comparing groups | C2 value | P value corrected by Bonferroni |
| --- | --- | --- |
| N vs ATP | -15 | 0.000 |
| ATP vs RH+ATP | 13 | 0.002 |
| ATP vs ME49+ATP | 8 | 0.045 |
| ATP vs VEG+ATP | 9.55 | 0.025 |
| RH+ATP vs ME49+ATP | -8.5 | 0.042 |
| RH+ATP vs VEG+ATP | -5.5 | 0.188 |
| ME49+ATP vs VEG+ATP | 3 | 0.473 |

**Figure 3-b1 C2=17.226, df=4, p=0.002**

**Figure 3-b2. C2=17.64, df=4, p=0.001**

| Comparing groups | C2 value | P value corrected by Bonferroni |
| --- | --- | --- |
| N vs ATP | -16 | 0.000 |
| ATP vs RH+ATP | 12 | 0.004 |
| ATP vs ME49+ATP | 9 | 0.02 |
| ATP vs VEG+ATP | 10 | 0.019 |
| RH+ATP vs ME49+ATP | -7 | 0.094 |
| RH+ATP vs VEG+ATP | -5 | 0.231 |
| ME49+ATP vs VEG+ATP | 2 | 0. 632 |

**Figure 4 C2=13.097, df=3, p=0.004**

| Comparing groups | C2 value | P value corrected by Bonferroni |
| --- | --- | --- |
| N vs ATP | -11 | 0.001 |
| ATP vs RH+ATP | 9 | 0.007 |
| ATP vs RH-∆*rop18*+ATP | 1.19 | 0.234 |
| RH+ATP vs RH-∆*rop18*+ATP | -7 | 0.037 |

**Figure-5b C2=8.831, df=2, p=0.012**

| Comparing groups | C2 value | P value corrected by Bonferroni |
| --- | --- | --- |
| Pos ctrl vs experiment | 3 | 0.238 |
| Neg ctrl vs experiment | -7.5 | 0.003 |

**Figure-6c C2=12.995, df=3, p=0.005**

| Comparing groups | C2 value | P value corrected by Bonferroni |
| --- | --- | --- |
| N vs ATP | -9.75 | 0.004 |
| ATP vs NF449 | 10 | 0.002 |
| ATP vs ATP+NF449 | 7.735 | 0.025 |
| NF449 vs ATP+NF449 | 6.875 | 0.04 |

| Comparing groups | C2 value | P value corrected by Bonferroni |
| --- | --- | --- |
| N vs ATP | -11.375 | 0.006 |
| ATP vs pcDNA3.1-P2X1+ATP | -9 | 0.03 |
| pcDNA3.1-P2X1+ATP vs pcDNA3.1-P2X1+ATP+pcDNA3.1-ROP18+ATP | 8.375 | 0.045 |

**Figure-6d C2=17.857, df=4, p=0.001**

**Figure 7b C2=13.254, df=3, p=0.004**

| Comparing groups | C2 value | P value corrected by Bonferroni |
| --- | --- | --- |
| N vs pcDNA3.1-P2X1+ATP | -11 | 0.001 |
| pcDNA3.1-P2X1+ATP vs pcDNA3.1-P2X1+ATP+pcDNA3.1-ROP18+ATP | 7 | 0.036 |

| Comparing groups | C2 value | P value corrected by Bonferroni |
| --- | --- | --- |
| N vs ATP | -15.95 | 0.002 |
| ATP vs RH+ATP | 26 | 0.000 |
| ATP vs RH-∆*rop18*+ATP | 3.05 | 0.559 |
| RH+ATP vs RH-∆*rop18*+ATP | -22.95 | 0.000 |

**Figure-8b C2=31.881, df=3, p=0.000**

**Figure-8d C2=11.925, df=3, p=0.008**

| Comparing groups | C2 value | P value corrected by Bonferroni |
| --- | --- | --- |
| N vs ATP | -10.75 | 0.001 |
| ATP vs RH+ATP | 8.25 | 0.014 |
| ATP vs RH-∆*rop18*+ATP | 4 | 0.234 |
| RH+ATP vs RH-∆*rop18*+ATP | -6.75 | 0.045 |

**Figure 9 c1 C2=12.981, df=3, p=0.005**

| Comparing groups | C2 value | P value corrected by Bonferroni |
| --- | --- | --- |
| N vs ATP | -10.75 | 0.001 |
| ATP vs RH+ATP | 9.25 | 0.006 |
| ATP vs RH-∆*rop18*+ATP | 1.5 | 0.655 |
| RH+ATP vs RH-∆*rop18*+ATP | -6.75 | 0.044 |

| Comparing groups | C2 value | P value corrected by Bonferroni |
| --- | --- | --- |
| N vs ATP | 8 | 0.017 |
| ATP vs RH+ATP | 8 | 0.017 |
| RH+ATP vs RH-∆*rop18*+ATP | 12 | 0.000 |

**Figure 9 c2 C2=14.138, df=3, p=0.003**

**Figure 9 c3 C2=10.739, df=3, p=0.013**

| Comparing groups | C2 value | P value corrected by Bonferroni |
| --- | --- | --- |
| N vs ATP | -4.375 | 0.192 |
| ATP vs RH+ATP | -9.5 | 0.005 |
| RH+ATP vs RH-∆*rop18*+ATP | 9.375 | 0.005 |

| Comparing groups | C2 value | P value corrected by Bonferroni |
| --- | --- | --- |
| N vs ATP | 8.25 | 0.014 |
| ATP vs RH+ATP | 11.75 | 0.000 |
| RH+ATP vs RH-∆*rop18*+ATP | 7.75 | 0.021 |

**Figure 9 c4 C2=13.087, df=3, p=0.003**

**Figure 9 c5 C2=14.118, df=3, p=0.003**

| Comparing groups | C2 value | P value corrected by Bonferroni |
| --- | --- | --- |
| N vs ATP | 4 | 0.235 |
| ATP vs RH+ATP | -12 | 0.000 |
| RH+ATP vs RH-∆*rop18*+ATP | 8 | 0.017 |

| Comparing groups | C2 value | P value corrected by Bonferroni |
| --- | --- | --- |
| ATP vs RH+ATP | 10.875 | 0.001 |
| RH+ATP vs RH-∆*rop18*+ATP | -8.125 | 0.016 |

**Figure 10 b1 C2=12.973, df=3, p=0.005**

| Comparing groups | C2 value | P value corrected by Bonferroni |
| --- | --- | --- |
| ATP vs RH+ATP | 8.25 | 0.014 |
| RH+ATP vs RH-∆*rop18*+ATP | -7.75 | 0.021 |

**Figure 10 b2 C2=13.909, df=3, p=0.003**

| Comparing groups | C2 value | P value corrected by Bonferroni |
| --- | --- | --- |
| ATP vs RH+ATP | 8 | 0.017 |
| RH+ATP vs RH-∆*rop18*+ATP | -6 | 0.045 |

**Figure 10 b3 C2=14.159, df=3, p=0.003**

| Comparing groups | C2 value | P value corrected by Bonferroni |
| --- | --- | --- |
| ATP vs RH+ATP | -0.802 | 0.0794 |
| RH+ATP vs RH-∆*rop18*+ATP | -2.375 | 0.479 |

**Figure 10 b4 C2=9.539, df=3, p=0.023**

| Comparing groups | C2 value | P value corrected by Bonferroni |
| --- | --- | --- |
| N vs ATP | -18.5 | 0.000 |
| ATP vs RH+ATP | 24.45 | 0.000 |
| ATP vs ME49+ATP | 24.2 | 0.000 |
| ATP vs VEG+ATP | 14.8 | 0.02 |
| RH+ATP vs ME49+ATP | -0.25 | 0.969 |
| RH+ATP vs VEG+ATP | -9.65 | 0.131 |
| ME49+ATP vs VEG+ATP | -9.4 | 0. 141 |

**Figure S1 b1 C2=29.915, df=4, p=0.000**

| Comparing groups | C2 value | P value corrected by Bonferroni |
| --- | --- | --- |
| N vs ATP | -26.9 | 0.000 |
| ATP vs RH+ATP | 20 | 0.001 |
| ATP vs ME49+ATP | 25.7 | 0.000 |
| ATP vs VEG+ATP | 26.9 | 0.000 |
| RH+ATP vs ME49+ATP | 5.7 | 0.337 |
| RH+ATP vs VEG+ATP | 6.9 | 0.245 |
| ME49+ATP vs VEG+ATP | 1.2 | 0. 84 |

**Figure S1 b2 C2=19.696, df=4, p=0.001**

| Comparing groups | C2 value | P value corrected by Bonferroni |
| --- | --- | --- |
| N vs ATP | -23.05 | 0.000 |
| ATP vs RH+ATP | 14 | 0.032 |
| ATP vs ME49+ATP | 18.65 | 0.004 |
| ATP vs VEG+ATP | 13.55 | 0.037 |
| RH+ATP vs ME49+ATP | 4.65 | 0.475 |
| RH+ATP vs VEG+ATP | -0.45 | 0.945 |
| ME49+ATP vs VEG+ATP | -5.1 | 0. 434 |

**Figure S2-b1 C2=14.125, df=4, p=0.007**

| Comparing groups | C2 value | P value corrected by Bonferroni |
| --- | --- | --- |
| N vs ATP | -24.278 | 0.000 |
| ATP vs RH+ATP | 23.95 | 0.000 |
| ATP vs ME49+ATP | 33.05 | 0.000 |
| ATP vs VEG+ATP | 16.3 | 0.012 |
| RH+ATP vs ME49+ATP | 9.1 | 0.16 |
| RH+ATP vs VEG+ATP | -7.65 | 0.238 |
| ME49+ATP vs VEG+ATP | -16.75 | 0. 01 |

**Figure S2-b2 C2=28.036, df=4, p=0.000**

| Comparing groups | C2 value | P value corrected by Bonferroni |
| --- | --- | --- |
| N vs ATP | -17.75 | 0.006 |
| ATP vs RH+ATP | 20.3 | 0.002 |
| ATP vs ME49+ATP | 24.4 | 0.000 |
| ATP vs VEG+ATP | 27.05 | 0.000 |
| RH+ATP vs ME49+ATP | 4.1 | 0.524 |
| RH+ATP vs VEG+ATP | 6.75 | 0.294 |
| ME49+ATP vs VEG+ATP | 2.65 | 0. 681 |

**Figure S3-b1 C2=21.834, df=4, p=0.000**

| Comparing groups | C2 value | P value corrected by Bonferroni |
| --- | --- | --- |
| N vs ATP | -20.75 | 0.001 |
| ATP vs RH+ATP | 22.05 | 0.001 |
| ATP vs ME49+ATP | 27.3 | 0.000 |
| ATP vs VEG+ATP | 24.15 | 0.000 |
| RH+ATP vs ME49+ATP | 5.25 | 0.413 |
| RH+ATP vs VEG+ATP | 2.1 | 0.743 |
| ME49+ATP vs VEG+ATP | -3.15 | 0.623 |

**Figure S3-b2 C2=22.809, df=4, p=0.000**

**Figure S4-b1 C2=13.172, df=3, p=0.004**

| Comparing groups | C2 value | P value corrected by Bonferroni |
| --- | --- | --- |
| N vs ATP | 7.125 | 0.034 |
| ATP vs RH+ATP | 11.125 | 0.001 |
| ATP vs RH-∆*rop18*+ATP | 8.875 | 0.008 |
| RH+ATP vs RH-∆*rop18*+ATP | -2.25 | 0.504 |

**Figure S4-b2 C2=13.257, df=3, p=0.004**

| Comparing groups | C2 value | P value corrected by Bonferroni |
| --- | --- | --- |
| N vs ATP | -12 | 0.000 |
| ATP vs RH+ATP | 7.25 | 0.031 |
| ATP vs RH-∆*rop18*+ATP | 7.25 | 0.031 |
| RH+ATP vs RH-∆*rop18*+ATP | 4.75 | 0.157 |
