# Supplementary figures and images for: Toxoplasma gondii ROP18 inhibits human glioblastoma cell apoptosis through a mitochondrial pathway by targeting host cell P2X1
Source: Parasit Vectors. 2019 Jun 4;12:284. doi: 10.1186/s13071-019-3529-1 (PMC6547611; doi:10.1186/s13071-019-3529-1)

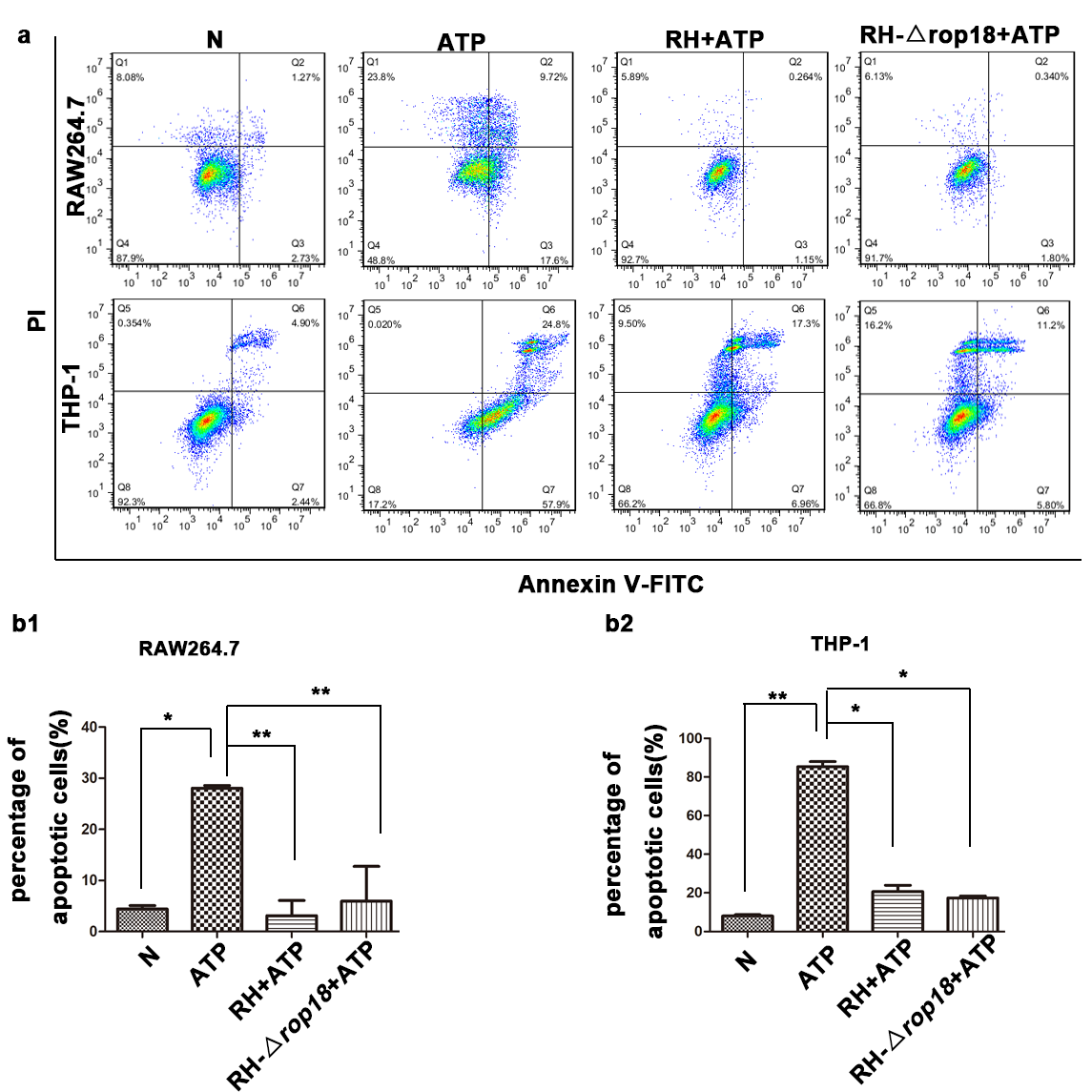

Supplement: Supplementary file 5 — Additional file 5: Figure S4. Effect of T. gondii virulence factor ROP18 on ATP-induced apoptosis of RAW264.7 and THP-1 cells. RAW264.7 and THP-1 cells were infected with RH or RH-Δrop18 tachyzoites (MOI = 13) or left uninfected to serve as the normal control (N) or positive control (ATP treatment). At 12 h post-infection, 1 mg/ml ATP was added to the cells for an additional 12 h, except in the normal control group. a Representative flow cytometry data. b Quantification of the flow cytometry data. The percentages of apoptotic cells were separately determined for each group of cells. The experiments were repeated four times for Kruskal–Wallis H-test statistical analysis (*P < 0.05, **P < 0.01). [file 13071_2019_3529_MOESM5_ESM.tif]

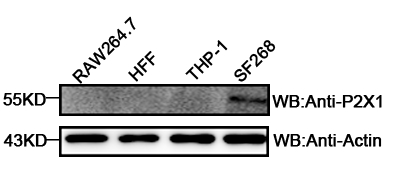

Supplement: Supplementary file 7 — Additional file 7: Figure S5. Western blot analysis of P2X1 in SF268, RAW264.7, HFF and THP-1 cells. RAW264.7, HFF, THP-1 and SF268 cells were grown in a T25 flask to 100% confluence and then harvested and lysed. Total proteins for each sample were subjected to SDS-PAGE and western blotting analysis with P2X1 antibody. [file 13071_2019_3529_MOESM7_ESM.tif]
