# Supplementary material for: Toxoplasma gondii ROP18 inhibits human glioblastoma cell apoptosis through a mitochondrial pathway by targeting host cell P2X1
Source: Parasit Vectors. 2019 Jun 4;12:284. doi: 10.1186/s13071-019-3529-1 (PMC6547611; doi:10.1186/s13071-019-3529-1)
Supplement: Supplementary file 6 — Additional file 6: Table S2. Summarized information for Toxoplasma gondii modulation of immune and neural cell apoptosis. [file 13071_2019_3529_MOESM6_ESM.doc]

| Cell line | Origin | Cell type | MOI | Genotype | Infection tim**e** | Apoptosis | Reference | |
| --- | --- | --- | --- | --- | --- | --- | --- | --- |
| HL-60  Jurkat  SKW6.4  THP-1  Jurkat  PBMCS  THP-1  Spleen  HL60  U937  HL60 | human  human  human  human  human  human  human  mouse  human  human  human | promyeloblast  T lymphocyte  B lymphocyte  monocyte  T lymphocyte  monocyte  monocyte  Spleen cells  promyeloblast  Lymphocyte  promyeloblast | 10:1 or 20:1  1:1  10:1 30:1 50:1  1:1 10:1 20:1  (0.05,1,10)×10^7^  1:1  1:1or 30:1  10:1  10:1  30:1  2:1 5:1 | NTE/RH  RH  NTE  RH  NTE  Tgctwh3  RH  RH  NTE  NTE  RH | 9h  16h  24h  11h  24h  6,12,24h  18h  18h  9h  9h  2,6,12h | inhibition  inhibition  inhibition  inhibition  inhibition  inhibition  inhibition  inhibition  inhibition  inhibition  inhibition | [1]  [2]  [3]  [4]  [5]  [6]  [7]  [8]  [9]  [9]  [10] |  |
| Jurkat | human | T lymphocyte | 30:1 | NTE | 24h | inhibition | [11] |  |
| Jurkat | human | T lymphocyte | 5:1-30:1 | NTE | 24h | inhibition | [12] |  |
| RAW  264.7 | mouse | macrophage | Transwell | NTE | 1,2,4,8,12,  24h | inhibition | [13] |  |
| CD^4+^,CD^8+^ | mouse | T lymphocyte | 1 × 10^5^ | ME49 | 3,6days | promotion | [14] |  |
| CD^4+^,CD^8+^ | mouse | T lymphocyte | cysts | ME49 | 7 days | promotion | [15] |  |
| N2a | mouse | neuroblast | Trans-well | Wh6 | 24h | promotion | [16] |  |
| C17.2 | mouse | neuroblast | Trans-well | Wh3, RH | 24h | promotion | [17] |  |

**Additional file 6: Table S2.** Summarized information of *Toxoplasma gondii* modulating immune and neuro cell apoptosis

**References**

1. Goebel S, Lüder CG, Gross U. Invasion by Toxoplasma gondii protects human-derived HL-60 cells from actinomycin D-induced apoptosis. Med Microbiol Immunol. 1999;187(4):221-6.

2. Yamada T, Tomita T, Weiss LM, Orlofsky A. Toxoplasma gondii inhibits granzyme B-mediated apoptosis by the inhibition of granzyme B function in host cells. INT J PARASITOL. 2011;41(6):595-607.

3. Vutova P, Wirth M, Hippe D, Gross U, Schulze-Osthoff K, Schmitz I, et al. Toxoplasma gondii inhibits Fas/CD95-triggered cell death by inducing aberrant processing and degradation of caspase 8. Cell Microbiol. 2007;9(6):1556-70.

4. Quan J, Cha G, Zhou W, Chu J, Nishikawa Y, Lee Y. Involvement of PI 3 kinase/Akt-dependent Bad phosphorylation in Toxoplasma gondii-mediated inhibition of host cell apoptosis. Exp Parasitol. 2013;133(4):462-71.

5. Keller P, Schaumburg F, Fischer SF, Hacker G, Gross U, Luder CG. Direct inhibition of cytochrome c-induced caspase activation in vitro by Toxoplasma gondii reveals novel mechanisms of interference with host cell apoptosis. Fems Microbiol Lett. 2006;258(2):312-9.

6. Cai Y, Chen H, Mo X, Tang Y, Xu X, Zhang A, et al. Toxoplasma gondii inhibits apoptosis via a novel STAT3-miR-17–92-Bim pathway in macrophages. Cell Signal. 2014;26(6):1204-12.

7. Hwang I, Quan JH, Ahn M, Hassan Ahmed HA, Cha G, Shin D, et al. Toxoplasma gondii infection inhibits the mitochondrial apoptosis through induction of Bcl-2 and HSP70. Parasitol Res. 2010;107(6):1313-21.

8. Kim JY, Ahn MH, Jun HS, Jung JW, Ryu JS, Min DY. Toxoplasma gondii inhibits apoptosis in infected cells by caspase inactivation and NF-kappaB activation. Yonsei Med J. 2006;47(6):862-9.

9. Goebel S, Gross U, Luder CG. Inhibition of host cell apoptosis by Toxoplasma gondii is accompanied by reduced activation of the caspase cascade and alterations of poly(ADP-ribose) polymerase expression. J Cell Sci. 2001;114(Pt 19):3495-505.

10. Angeloni MB, Silva NM, Castro AS, Gomes AO, Silva DAO, Mineo JR, et al. Apoptosis and S Phase of the Cell Cycle in BeWo Trophoblastic and HeLa Cells are Differentially Modulated by Toxoplasma gondii Strain Types. Placenta. 2009;30(9):785-91.

11. Hippe D, Lytovchenko O, Schmitz I, CG L. Fas/CD95-Mediated Apoptosis of Type II Cells Is Blocked by Toxoplasma gondii Primarily via Interference with the Mitochondrial Amplification Loop. Infect Immun. 2008;76(7):2905-12.

12. Graumann K, Schaumburg F, Reubold T, Hippe D, Eschenburg S, Lüder C. Toxoplasma gondii inhibits cytochrome c-induced caspase activation in its host cell by interference with holo-apoptosome assembly. Microb Cell. 2015;2(5):150-62.

13. Gais A, Beinert N, Gross U, Lüder CGK. Transient inhibition of poly(ADP-ribose) polymerase expression and activity by Toxoplasma gondii is dispensable for parasite-mediated blockade of host cell apoptosis and intracellular parasite replication. Microbes Infect. 2008;10(4):358-66.

14. Begum-Haque S, Haque A, Kasper LH. Apoptosis in Toxoplasma gondii activated T cells: The role of IFNγ in enhanced alteration of Bcl-2 expression and mitochondrial membrane potential. Microb Pathgenesis. 2009;47(5):281-8.

15. Liesenfeld O, Kosek JC, Suzuki Y. Gamma interferon induces Fas-dependent apoptosis of Peyer's patch T cells in mice following peroral infection with Toxoplasma gondii. Infect Immun. 1997;65(11):4682-9.

16. Zhang YH, Chen H, Chen Y, Wang L, Cai YH, Li M, et al. Activated microglia contribute to neuronal apoptosis in Toxoplasmic encephalitis. Parasit Vectors. 2014;7:372.

17. Zhou J, Gan X, Wang Y, Zhang X, Ding X, Chen L, et al. Toxoplasma gondii prevalent in China induce weaker apoptosis of neural stem cells C17.2 via endoplasmic reticulum stress (ERS) signaling pathways. Parasit Vectors. 2015;8(1):73.
